# Anxiolytic effect of YangshenDingzhi granules: Integrated network pharmacology and hippocampal metabolomics

**DOI:** 10.3389/fphar.2022.966218

**Published:** 2022-10-31

**Authors:** Shimeng Lv, Weibo Dai, Yan Zheng, Ping Dong, Yihong Yu, Yifan Zhao, Shiguang Sun, Dezhong Bi, Chuanguo Liu, Fabin Han, Jibiao Wu, Tingting Zhao, Yuexiang Ma, Feng Zheng, Peng Sun

**Affiliations:** ^1^ School of Traditional Chinese Medicine, Shandong University of Traditional Chinese Medicine, Jinan, China; ^2^ Department of Pharmacy, Zhongshan Hospital of Traditional Chinese Medicine, Zhong Shan, China; ^3^ Research Center of Translational Medicine, Central Hospital Affiliated to Shandong First Medical University, Jinan, China; ^4^ School of Management, Shandong University of Traditional Chinese Medicine, Jinan, China; ^5^ School of Pharmacy, Shandong University of Traditional Chinese Medicine, Jinan, China; ^6^ The Second Affiliated Hospital of Shandong University of Traditional Chinese Medicine, Jinan, China; ^7^ Experimental Center, Shandong University of Traditional Chinese Medicine, Jinan, China; ^8^ Innovative Institute of Chinese Medicine and Pharmacy, Shandong University of Traditional Chinese Medicine, Jinan, China; ^9^ School of Foreign Language, Shandong University of Traditional Chinese Medicine, Jinan, China; ^10^ Department of Neurosurgery, The Second Affiliated Hospital of Fujian Medical University, Quanzhou, China

**Keywords:** network pharmacology, metabolomics, anxiolytics, YangshenDingzhi granules, molecular docking, molecular dynamics simulation

## Abstract

Anxiety disorder is one of the most common mental diseases. It is mainly characterized by a sudden, recurring but indescribable panic, fear, tension and/or anxiety. Yangshendingzhi granules (YSDZ) are widely used in the treatment of anxiety disorders, but its active ingredients and underlying mechanisms are not yet clear. This study integrates network pharmacology and metabolomics to investigate the potential mechanism of action of YSDZ in a rat model of anxiety. First, potential active ingredients and targets were screened by network pharmacology. Then, predictions were verified by molecular docking, molecular dynamics and western blotting. Metabolomics was used to identify differential metabolites and metabolic pathways. All results were integrated for a comprehensive analysis. Network pharmacology analysis found that Carotene, β-sitosterol, quercetin, Stigmasterol, and kaempferol in YSDZ exert anxiolytic effects mainly by acting on IL1β, GABRA1, PTGS1, ESR1, and TNF targets. Molecular docking results showed that all the affinities were lower than −5 kcal/mol, and the average affinities were −7.7764 kcal/mol. Molecular dynamics simulation results showed that RMSD was lower than 2.5 A, and the overall conformational changes of proteins were small, indicating that the small molecules formed stable complexes with proteins. The results of animal experiments showed that YSDZ exerts anxiolytic effects by regulating GABRA1 and TNF-α, ameliorating pathological damage in hippocampal CA1, and regulating metabolic pathways such as thiamine, cysteine and methionine metabolism, lysine biosynthesis and degradation. Altogether, we reveal multiple mechanisms through which YSDZ exerts its anti-anxiety effects, which may provide a reference for its clinical application and drug development.

## Introduction

**FIGURE 1:**
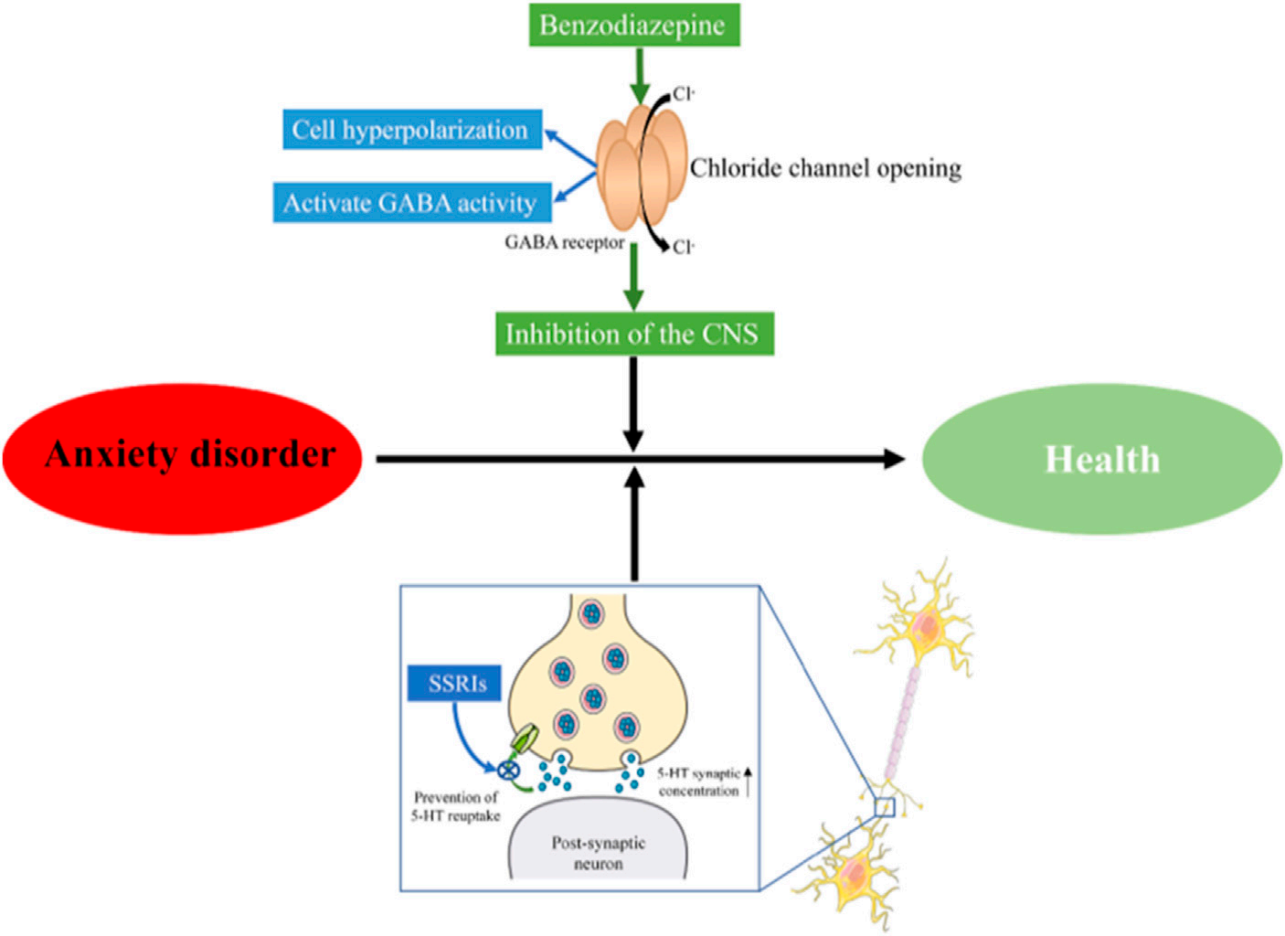
Mechanism of antianxiety action. CNS, Central Nervous System; SSRIs, selective serotonin reuptake inhibitors.

Anxiety disorder is a mental health issue characterized by the sudden and recurring but indescribable panic, fear, tension, and/or anxiety, accompanied by palpitations, sweating, and other autonomic nervous system disorders. Its worldwide prevalence is estimated to be 3.8%–25%, while the prevalence of chronic diseases is estimated to be as high as 70% (Remes et al., 2016). Anxiety disorder is one of the most common mental health problems and impacts the quality of life and social stability (Kandola et al., 2018). The main treatments for anxiety in western medicines include selective serotonin reuptake inhibitors (SSRIs), benzodiazepines (diazepam), and so on (Stonerock et al., 2105; Amorim et al., 2018). Benzodiazepines act on γ-aminobutyric acid (GABA) receptors, enhance GABA activity, further open chloride ion channels, and make chloride ions enter cells in large quantities, causing hyperpolarization of nerve cells, thereby playing a central inhibitory effect and treating anxiety (Cornett et al., 2018). The mechanism of action of SSRIs is to inhibit the reuptake of 5-HT by the presynaptic membrane, increase the concentration of 5-HT in the synaptic cleft, improve the conduction of 5-HT neurons, and exert anxiolytic effects (Bandelow et al., 2017) (Figure 1). However, the long-term use of these medications can result in drug dependence, memory and cognitive impairment, and increase the risk of motor impairment (Geng et al., 2021). Moreover, patients often experience delayed effects, high rates of non-response, nausea and headache in the adminstration of the above-mentioned drugs (Qu et al., 2021; Wei et al., 2022; Wang et al., 2019). Therefore, there has been a necessity to develop anti-anxiety drugs with fewer side effects currently.

Traditional Chinese medicine (TCM) has been characterized by compounds of multiple complexes that target multiple pathways (Zhang et al., 2019). Previous evidence has shown that TCM has reduced side effects, can be individualized, and exhibits a considerable clinical research value (Li et al., 2021). Indeed, some TCMs were suggested as the treatment for anxiety (Liu et al., 2015). Therefore, in order to reduce the side effects and adverse reactions caused by the treatment of western medicine, researchers have been investigating the use of TCM to treat anxiety. YangshenDingzhi Granules (YSDZ) have been recognized as a TCM compound, which is composed of *Rehmanniae Radix Praeparata*, *Polygala tenuifolia Willd*, *Poria cocos (Schw.) Wolf*, *Saposhnikovia divaricata (Trucz.) Schischk*, *Cyrtomium fortunei J. Sm.,Talinum paniculatum (Jacq.) Gaertn.,Chrysanthemum indicum L., Aurantii Fructus*. It has been reported that the extract of *Chrysanthemum indicum L.* has neuroprotective effect, which can inhibit the apoptosis of neurons and the excessive response of neuroinflammation (Yan et al., 2021). *Aurantii Fructus.* has a rapid antidepressant effect that is dependent on the PKA/CREB/BDNF pathway and subsequently modulates downstream synaptic transmission (Wu et al., 2021). And there is ingredient evidence that Chinese herbal compounds or extracts of *Rehmanniae Radix Praeparata, Polygala tenuifolia Willd, and Poria cocos (Schw.) Wolf* have anxiolytic effects (Li et al., 2021; Ning et al., 2021; Wu et al., 2018). YSDZ has been proven to improve anxiety in patients; however, the active components of YSDZ and their potential anti-anxiety mechanisms have been not clear yet.

Network pharmacology integrates and organizes complex networks between drugs, targets and diseases (Wang et al., 2021; Wu et al., 2022; Jia et al., 2021). It focuses on high-throughput screening, network visualization, and analysis, which are beneficial features to the development of TCM. Besides, molecular docking is a method widely used in drug research (Pinzi et al., 2019), which can study the function and mechanism of drugs by predicting their binding mode and binding free energy with proteins of interest. It can study the action and mechanism of drugs by predicting the binding mode of drugs and the free energy of binding to target proteins, and is widely used in central nervous system diseases such as Alzheimer’s disease, anxiety, and depression (Jabir et al., 2021; Wu et al., 2022; Shi et al., 2021). Virtual screening based on molecular docking methods has become a necessary process for drug development (Dong et al., 2018). In parallel, molecular dynamics (MD) is a molecular simulation method that integrates physical, mathematical and chemical techniques (Hollingsworth et al., 2018), and helps to study the motion process of proteins by tracking changes in protein conformation over time (Collier et al., 2020). Metabolomics consists of the analysis of metabolites in biological cells or tissues aims to identify abnormal metabolic networks relavant to diseases. The identified metabolites are used to delineate the changes within metabolic pathways, and then elucidate the response mechanism to different stimuli (Johnson et al., 2016). Previous studies have proved that the combination of network pharmacology and metabolomics is an effective way to elucidate the treatment of affective disorders (such as anxiety and depression) by TCM (Qu et al., 2021; Liu et al., 2021). Meanwhile, this approach compensates the lack of experimental validation in network pharmacology and the lack of upstream molecular mechanisms and drug binding targets in metabolomics approaches (Li et al., 2021).

Chronic stress is a key factor in the pathogenesis of psychiatric diseases such as anxiety disorder. Therefore, animal models of chronic restraint stress (CRS) are often used to simulate anxiety-like behaviors (Liu et al., 2020). In this study, the main compounds of YSDZ and their potential targets relevant for anxiety intervention were predicted with a network pharmacology method for the first time. Molecular docking and molecular dynamics verified ligand-protein interactions. Then, the anti-anxiety effect of YSDZ was verified in CRS-induced anxiety rats, while predicted targets were assessed by Western blot (WB) and Immunofluorescence double labeling. Finally, we performed the non-targeted metabolomics of the hippocampus of these rats to further clarify the underlying mechanisms of the anxiolytic actions of YSDZ. This report may provide a scientific basis to guide the development of novel drugs.

## Experimental materials and methods

### Animal experiments

Twenty-four male Wistar rats (weight 200–220 g, 6–8 weeks old) were selected from China Jinan Pengyue Laboratory Animal Breeding Co., Ltd. Animals were adaptively fed with circadian inversion for 1 week under 21°C ± 1°C, 55% relative humidity, 12/12 h light/dark cycles and food and water *ad libitum*. Next, animals were divided into 6 groups: Control, model (CRS), Yangshen Dingzhi (CRS + YSDZ, 2, 4, 8 g/kg), and Diazepam (CRS + Diazepam, 1.38 mg/kg) groups. YSDZ (batch number: 210,912, Affiliated Hospital of Shandong University of Traditional Chinese Medicine, Jinan, Shandong) and diazepam (batch number: 210,401, Shandong Xinyi Pharmaceutical Company, Dezhou, Shandong) were dissolved in distilled water. Control and CRS groups received the same volume of the distilled water without drugs. The experimental procedures in this study were in compliance with the United States Public Health Administration’s Humane Management and Use of Laboratory Animals Policy, revised by the National Institutes of Health (NIH) in 2015. Experimental animal production license numbers: SCXK (Lu) 20190003 and SYXK (Lu) 2017-0022. All the experiments were approved by the ethics review committee of Shandong University of Traditional Chinese Medicine (No. DWSY201707025) and carried out in accordance with “the Guidelines for the Care and Use of Experimental Animals of the National Institutes of Health”.

### Chronic restraint stress

As a non-invasive stimulus, CRS is widely used to study anxiety-related behaviors (Liu et al., 2020; Moreno-Martínez et al., 2022; Vorvul et al., 2022). CRS group, YSDZ group (2, 4, 8 g/kg) and diazepam group were subjected to CRS for 21 days. A transparent plastic tube (5 cm height; 5.5 cm inner diameter; 22 cm length; proper space: 522.4 cm^3^) was used to cause restraint stress, and the tube length was adjusted according to body weight. In the restrained state, rats were allowed to breathe freely but were not allowed to move through the evenly distributed vents on the plastic tube. Rats were confined to the tubes in their usual home cages and kept in a supine position for 6 h a day (from 9:00 to 15:00, after drug intervention). Rats in the control group were transferred to the same room for the same time duration every day, but were not subjected to CRS (Geng et al., 2021).

### Open field test

Open field experiments were carried out at night on the second day after drug treatment (experimental day 29) and CRS. A dim red light (<12lux) was turned on in a quiet environment and the skin on the back neck of the rat was pinched. The rat was then placed in the center of a black open field box (100 cm × 100 cm) with its back to the researcher and was allowed to move freely. The area of the open field box was divided into nine grids, in which the central area accounted for 1/9 of the entire open field and the rest was the outer peripheral area. The SuperMaze + high-throughput animal behavioral analysis software was used to record and track the total distance, the central area distance, average speed wandered by rats within 6 min (Chopade et al., 2020).

### Light-dark box test

The LDB test was carried out 1 day after OFT (experimental day 30) in a box consisting of a dark and a bright room (25 cm × 25 cm × 30 cm; the box was separated by a door of 6.5 cm × 6.5 cm). The experiment was also carried out at night. Rats were placed in the center of the illuminated chamber and allowed to wonder for 5 min. The SuperMaze + high-throughput tracking system was used to record the behavior of rats, and analyze their total distance, light area distance, time in the light area and light area entrance (Chopade et al., 2021; Chopade et al., 2020).

### Elevated plus maze test

EPM was performed on experimental day 31 using a black box consisting of 2 open arms (10 cm × 50 cm) and 2 closed arms (10 cm × 50 cm), which are perpendicular to each other and cross to form a central area. The instrument was raised to 76 cm above the ground, and the rats were placed on the central platform with their heads facing the outstretched arms. The SuperMaze + high-throughput tracking system was used to record the behavior of rats for 5 min. The open arm entry time (OE), closed arm entry time (CE), time in the open arm (OT), time in the closed arm (CT) were analyzed. Based on these results, OT% and OE% were calculated according to the formulas: OT% = OT/(OT + CT)×100%; OE% = OE/(OE + CE)×100% (Chopade et al., 2020).

### Sample collection

After the behavioral experiment, rats were anesthetized with isoflurane, and the hippocampal tissues of some rats in each group were isolated by blunt dissection in a low temperature environment. After weighing, the hippocampus was dispensed into cryopreservation tubes, sealed with parafilm, and stored at −80°C until processing. The whole brains of the remaining rats in each group were fixed in 4% paraformaldehyde (PFA) solution and prepared for tissue slicing.

### Western blot

The rat hippocampus was homogenized and washed for 2-3 times with cold phosphate buffered saline (PBS) to remove blood stains. It was then cut into small pieces and placed in a homogenization tube with 1-2 small magnetic beads of 2 mm. A 10x buffer reagent (G2007; Servicebio; Wuhan, China) was added and the tissue was homogenized. The tube with the homogenized sample was then placed on ice for 30 min, shaken every 5 min to ensure the complete lysis of the tissue, and then centrifuged at 12,000 rpm for 10 min to collect the supernatant. Total protein concentration was determined using a BCA kit (G2026; Servicebio; Wuhan, China) following the manufacturer’s instructions. Proteins were subjected to sodium dodecyl sulfate polyacrylamide gel electrophoresis (SDS-PAGE) (G2003; Servicebio; Wuhan, China), transferred to a polyvinylidene fluoride (PVDF) membrane, blocked with 5% bovine serum albumin (BSA) for 1 h, and incubated with primary antibody overnight at 4°C. The primary antibodies were as follows: ACTIN (GB12001; Mouse; 1:2,000; Servicebio; Wuhan, China); TNF-α (GB113968; Rabbit; 1:1,000; Servicebio; Wuhan, China); GABAα1 (GB11402; Rabbit; 1:500; Servicebio); Wuhan, China). The secondary antibodies used were HRP-goat anti-mouse (GB25301; 1:5,000; Servicebio; Wuhan, China) and HRP-goat anti-rabbit (GB23303; 1:5,000; Servicebio; Wuhan, China). Finally, the bands were revealed through chemiluminescence, and the optical density values of target bands were analyzed with the Alpha software processing system.

### Hematoxylin-eosin stainings

First, The brain samples fixed in 4% PFA solution were taken, dewaxed and hydrated, washed with 0.1 M PBS three times, then immersed in hematoxylin dye for 5 min, washed with tap water after removal, differentiated with 1% hydrochloric acid alcohol for several seconds, returned to blue with 0.6% ammonia, and rinsed with running water for 5 s. They were dyed in eosin solution for 2 min and rinsed with running water. Finally, the slices were successively placed in gradient alcohol and xylene, dehydrated, transparent, and sealed with neutral gum.

### Immunofluorescence double labeling

Paraffin sections were obtained from the brain tissue of each rat. Each section was double-stained with anti-TNF-α (GB11188; 1:1000; Servicebio; Wuhan, China) and anti-GABAα1 (GB11402; 1:100; Servicebio; Wuhan, China). The staining steps of immunofluorescence double staining are briefly described as follows: First deparaffinize and rehydrate the paraffin sections, then antigen retrieval, circle and block endogenous peroxidase, block with serum. And then throw away the blocking solution slightly. Incubate slides with the first primary antibody (diluted with PBS appropriately) overnight at 4°C, placed in a wet box containing a little water. Then, samples were washed with PBS for 3 min × 5 min and incubated with the corresponding secondary antibody. The autofluorescence quenching agent was added, and the nucleus was restained with DAPI (G1012; Servicebio; Wuhan, China) and covered with antifadefluorescent mounting medium (G1401; Servicebio; Wuhan, China). Finally, the sections were observed with fluorescence microscope (Nikon Eclipse C1, NIKON, Japan).

### Statistical analysis

The above experimental data were analyzed and plotted by GraphPad Prism 8 software. Comparisons among multiple groups were tested by one-way analysis of variance (ANOVA). Results are expressed as mean ± standard error of the (Mean ± SEM), and *p* < 0.05 indicated that the groups were statistically different.

### Network pharmacology experiment

The Traditional Chinese Medicine Systems Pharmacology (TCMSP) database and analysis platform (http://tcmspw.com/tcmsp.php) was used for data collection. An oral bioavailability (OB) ≥ 30% and drug-likeness (DL) ≥ 0.18 were set as screening criteria to obtain active compounds in *Saposhnikovia divaricata (Trucz.) Schischk, Cyrtomium fortunei J. Sm., Talinum paniculatum (Jacq.) Gaertn., Rehmanniae Radix Praeparata, Chrysanthemum indicum L and Aurantii Fructus* as well as their predicted targets. Using the BATMAN-TCM database (http://bionet.ncpsp.org/batman-tcm/; screening critera score ≥ 20, *p*-value ≤ 0.05) (Qu et al., 2021) we obtained data on the active compounds of *Polygala tenuifolia Willd and Poria cocos (Schw.) Wolf* and their predicted targets. Through the Genecard [(www.genecards.org), Relevance Score > 20], OMIM (http://omim.org), and Drugbank (http://go.drugbank.com) databases, we entered “Anxiety Disorders” as the keyword to obtain targets related to anxiety disorders (Liu et al., 2021).

After intersecting the predicted targets of YSDZ and those of anxiety disorder, potential therapeutic targets were obtained and imported into the STRING database (http://www.string-db.org). We downloaded data on the interaction between potential human target proteins. At the same time, herbs, compounds and potential targets were imported into the Cytoscape software to construct a network diagram. The NetworkAnalyzer module function was used to calculate the topological parameters of each node within the network to find potential core compounds and potential core targets. The potential targets were uploaded to the CluoGO plug-in of Cytoscape to conduct gene ontology (GO) enrichment analysis, which includes biological process (BP), molecular function (MF), and cellular component (CC) analysis (Show pathways with pV ≤ 0.00001). Afterwards, KEGG enrichment analysis using Metascape (http://metascape.org) database."*Homo sapiens*” was selected as the species and personalized analysis was selected, with a *p*-value set to <0.01, a minimum count of 3, and an enrichment factor >1.5.

### Molecular dockings and molecular dynamics simulations

The potential core compounds and targets were screened in the above steps for molecular docking verification. First, small molecule structures were retrieved from PubChem (https://pubchem.ncbi.nlm.nih.gov/), optimized, and saved using Chem3D. Next, the crystal structures of target proteins were downloaded from the Protein Data Bank (PDB) database (https://www.rcsb.org/structure/). The Glide module (Maestro11.9) in the Schrödinger Maestro software was used to complete the virtual screening processing and optimization, while the Protein Preparation Wizard module was used for protein preparation. Preprocessing, optimization, and minimization of receptors were performed using the OPLS3e force field for constraint minimization (Mali et al., 2022).

The ligand with the best affinity for each target in molecular docking was further analyzed by MD simulations using the software Desmond version 2020. The molecular force field was selected as OPLS3e, and the 3-point interaction potential model (TIP3) water model was used. The charge of the system was neutralized by ion addition. Energy minimization of the entire system was achieved using the OPLS3e force field (all-atomic force field). The geometric structure of water molecules, bond lengths, and bond angles of heavy atoms were constrained by the SHAKE algorithm. The continuous system was simulated by applying periodic boundary conditions, and long-range static electricity was maintained by the particle grid Ewald method. The NPT ensemble was used with a temperature of 300 K and pressure of 1.0 bar to balance the system. The temperature-pressure parameter coupling adopted the Berendsen coupling algorithm. A 100-ns operation was performed with a time step of 1.2 fs and data were recorded every 100 ps and a total of 1,000 frames were recorded. Finally, the root mean square deviation (RMSD) of the main chain atoms were calculated and graphics were constructed to understand the nature of protein-ligand interactions (Friesner et al., 2004; Halgren et al., 2004; Friesner et al., 2006).

## Metabolomics analysis

### Metabolite extraction

A total of 25 mg of rat hippocampus was mixed into 500 μl of extraction solution (methanol: acetonitrile: water = 2: 2: 1 (V/V), containing isotope-labeled internal standard mixture), grinded at 35 Hz for 4 min and subjected to ultrasonic sonication for 5 min (in an ice water bath). These steps were repeated 2 or 3 times and the samples were left at −40°C for 1 h. Samples were centrifuged at 12,000 rpm (centrifugal force 13,800 (×g), radius 8.6 cm), 4°C, for 15 min and the supernatant was collected. An equal amount of supernatant was collected from all samples and mixed into a quality control (QC) sample.

### Detection conditions

In this study, the target compounds were chromatographed on a Waters ACQUITY UPLC BEH Amide (2.1 mm × 100 mm, 1.7 μm) LC column using a Vanquish (Thermo Fisher Scientific) ultra-high performance liquid chromatography (LC). phase A of LC was an aqueous phase containing 25 mmol/L ammonium acetate and 25 mmol/L ammonia water, while phase B was acetonitrile. The sample pan temperature was set at 4°C and the injection volume was set at 2 μl. The Thermo Q Exactive HFX mass spectrometer is capable of data acquisition of the primary and secondary mass spectrometry (MS) under the control software (Xcalibur, Thermo). Detailed parameters were as follows: sheath gas flow rate of 30 Arb, aux gas flow rate of 25 Arb, capillary temperature of 350°C, full ms resolution of 60,000, MS/MS resolution of 7,500, collision energy of 10/30/60 in NCE mode, spray voltage of 3.6 kV (positive) or −3.2 kV (negative).

### Data processing

**FIGURE 2:**
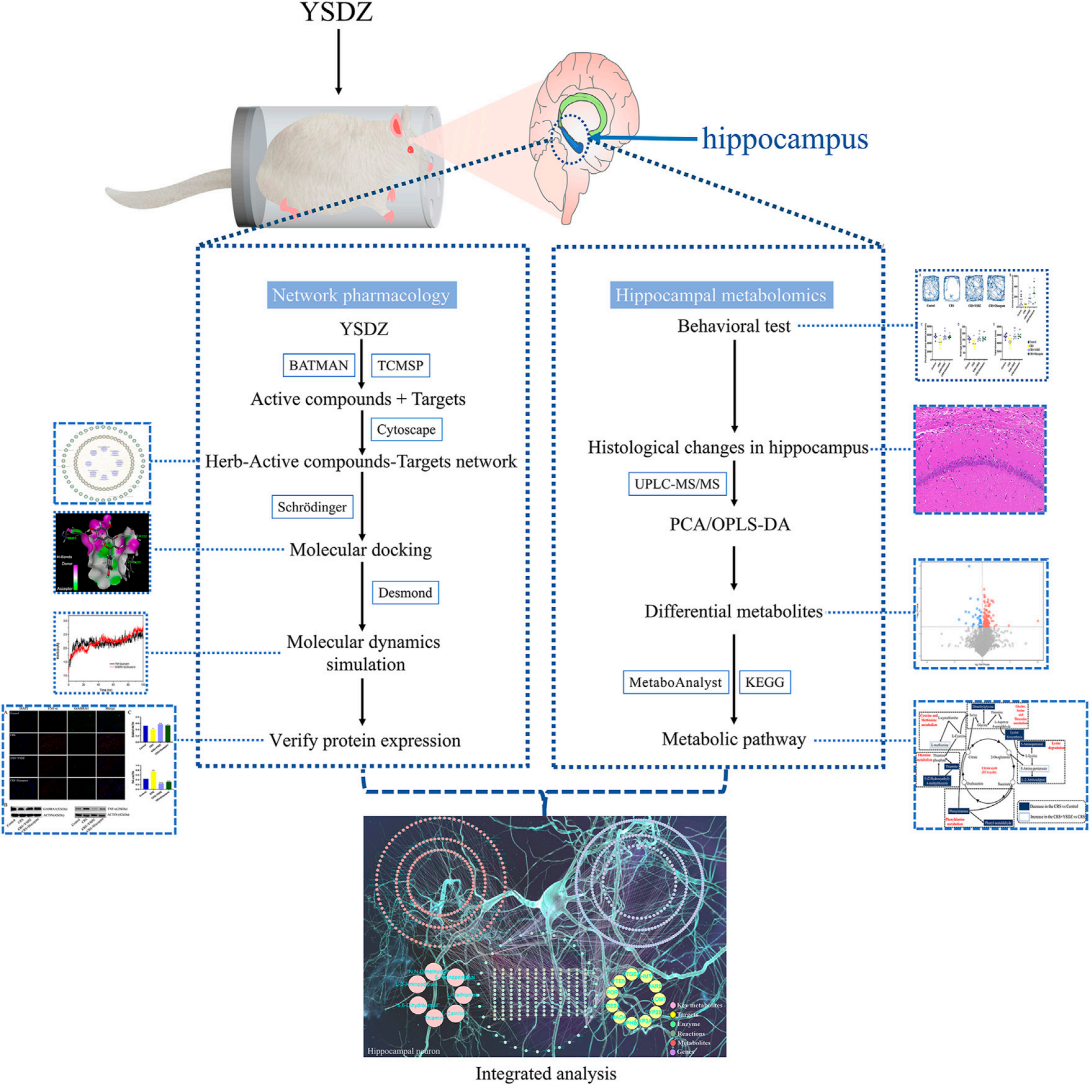
Framework of the study.

The raw data was converted into mzXML format by the ProteoWizard software, and the self-written *R* package (the kernel was XCMS) was used for peak identification, peak extraction, peak alignment and integration. These peaks were matched with the BiotreeDB (V2.1) self-built secondary mass spectrometry database for substance annotation. The cutoff value of the algorithm scoring was set to 0.3. After normalizing, the data was imported into SIMCA 14.1 (V16.0.2, Sartorius Stedim Data Analytics AB, Umea, Sweden). Principal component analysis (PCA) and orthogonal partial least squares-discriminant analysis (OPLS-DA) were performed after data standardization. Differential metabolites with a variable importance in the projection (VIP) greater than 1 and Student’s *t*-test (*p*-value) lower than 0.05 were used for metabolic pathway analysis (the research framework is shown in Figure 2).

## Result

### Yangshendingzhi granules improved anxiety-like behavior in rats

**FIGURE 3:**
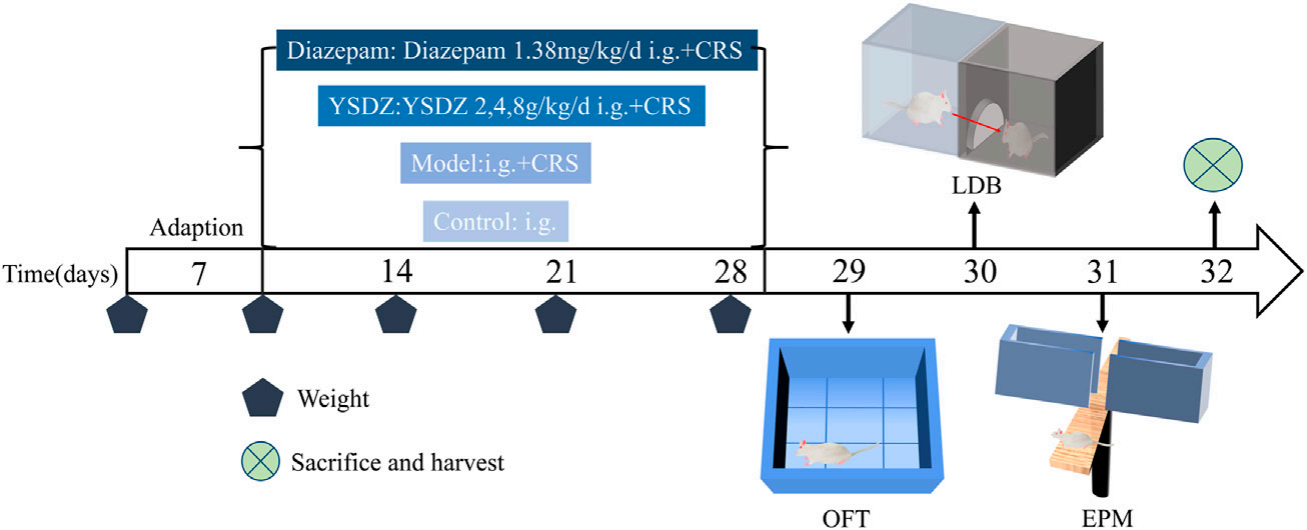
Experimental procedure.

**FIGURE 4:**
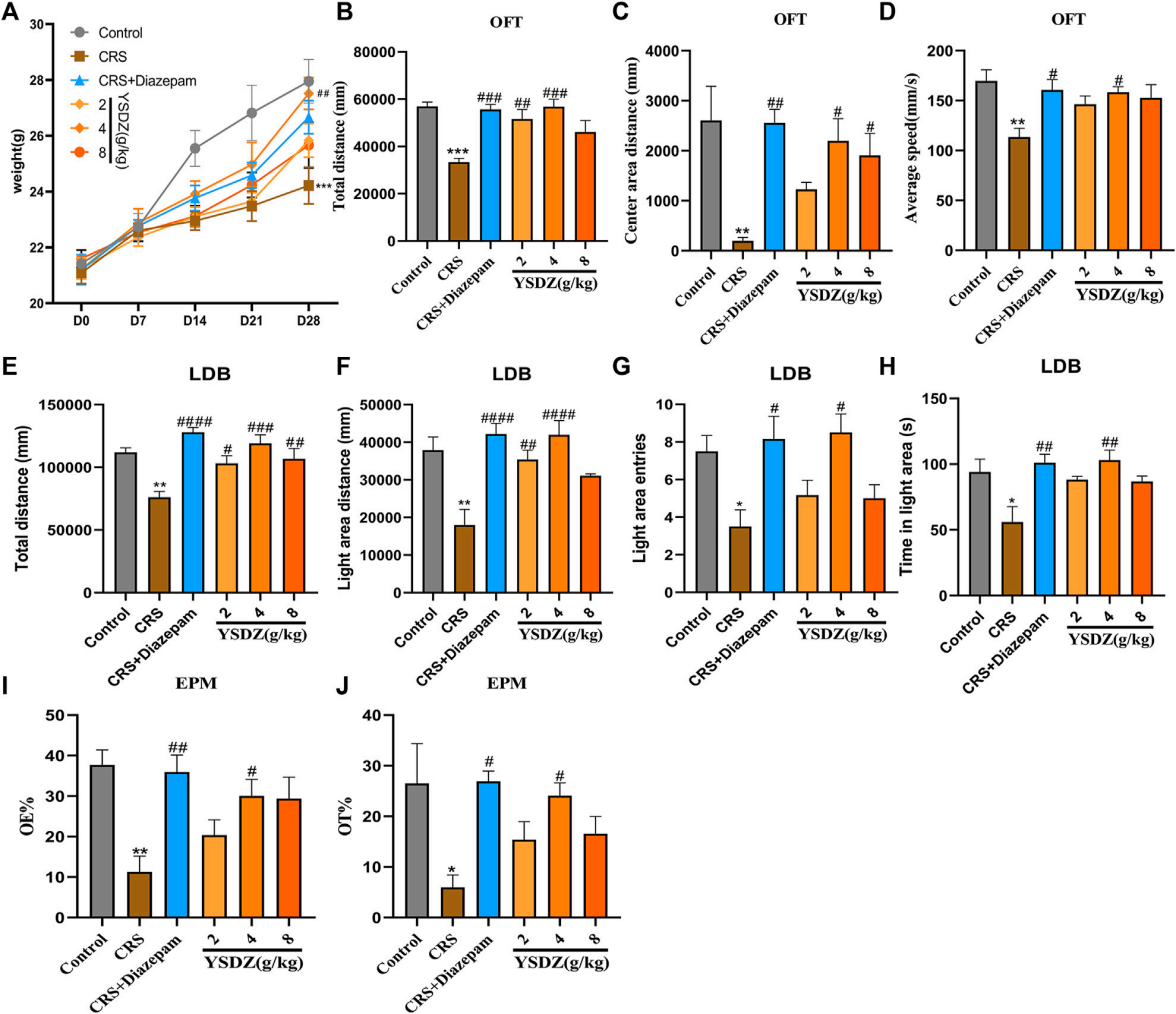
Body weight and behavior test. **(A) **Body weight. **(B) **Total distance (mm); **(C)** Center area distance (mm); **(D)** Average speed (mm/s); **(E)** Total distance (mm); **(F)** Light area distance (mm); **(G)** Light area entries; **(H)** Time in light area (s); **(I)** OE%; **(J)** OT%.*p < 0.05, **p < 0.01, ***p < 0.001, compared with Control; #*p* < 0.05, ##*p* < 0.01, ###*p* < 0.001, ####*p* < 0.0001, compared with CRS *via* one-way ANOVA.

Details about the protocol framework for the CRS intervention and behavioral analyses are shown in Figure 3. Rats under CRS stress showed a significant decrease in body weight on the 28th day when compared to the blank control group (*p* < 0.001). Meanwhile, YSDZ (4 g/kg) intervention could alleviate CRS-induced weight loss (*p* < 0.01) (Figure 4A). OFT results showed that CRS group induced a significant decrease in the centre area distance (*p* < 0.01) compared to that in the control group. This was prevented by the diazepam (*p* < 0.01) and YSDZ (4.8 g/kg, *p* < 0.05). Compared with the Control group, CRS significantly reduced the total distance (*p* < 0.001), and the intervention of diazepam (*p* < 0.001) and YSDZ (2.4 g/kg, *p* < 0.01, *p* < 0.001) could alleviate the above phenomenon. At the same time, diazepam (*p* < 0.05) and YSDZ (4 g/kg, *p* < 0.05) also reversed the CRS-induced decrease in average speed (*p* < 0.01) (Figures 4B–D).

Results of LDB showed that compared to the control group, CRS group showed significantly decreased in total distance (*p* < 0.01) and light area distance (*p* < 0.01), light area entries (*p* < 0.05) and time in the light area (*p* < 0.05). Following treatment with YSDZ and diazepam, these alterations were reverted to control levels (Figures 4E–H). In addition, results of EPM showed that OE% (*p* < 0.01) and OT% (*p* < 0.05) of the CRS group were lower than those of the control group. Interestingly, YSDZ (4 g/kg) and diazepam improved both OE% (*p* < 0.05, *p* < 0.01) and OT% (*p* < 0.05, *p* < 0.05). (Figures 4I,J). Behavioral results indicated that a successful and reliable model of anxiety was established using CRS, and that YSDZ induced anxiolytic effects similar to those of diazepam. At the same time, based on the above behavioral test results, we choose the optimal dose of YSDZ (4 g/kg) to enter the next experiment.

### Yangshendingzhi granules alleviates neuropathic injury in hippocampus

**FIGURE 5:**
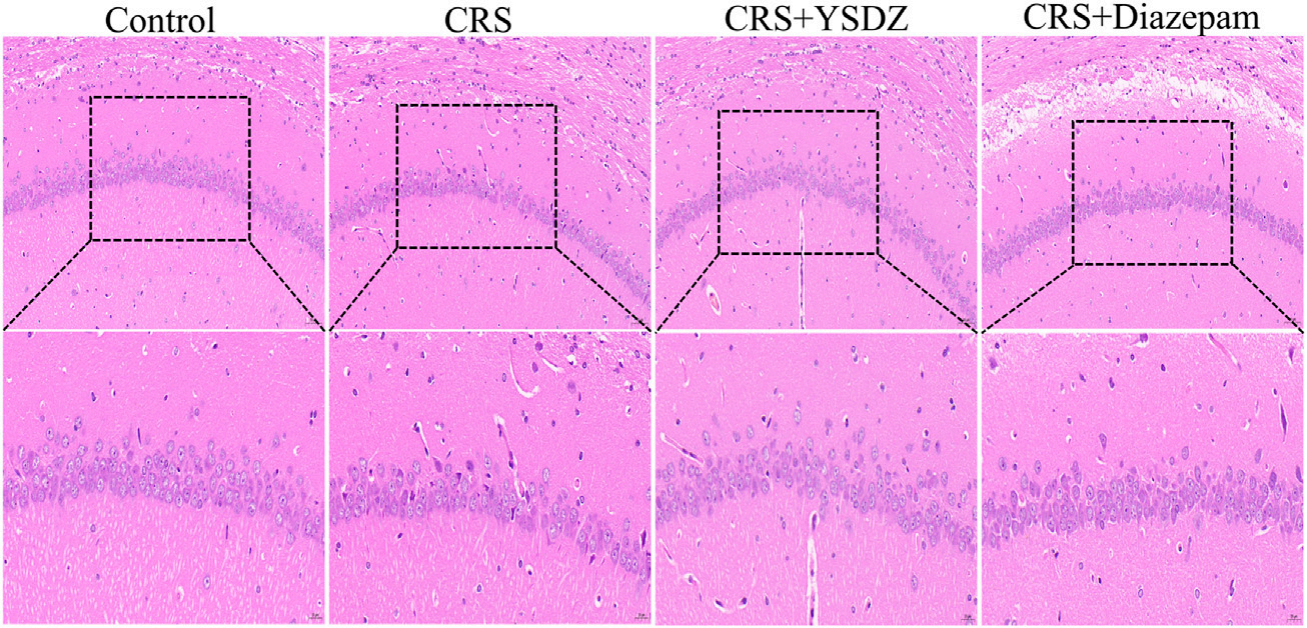
Histological changes in hippocampus.

HE staining results showed that regular morphology neurons in control group hippocampal with large and round nuclei, uniform light blue or blue, obvious nucleoli, and abundant cytoplasm. However, after performing CRS, the number of neurons in the hippocampal CA1 were slightly reduced, and the neurons were shriveled and stained, cell body was shrunk and deformed, the boundary between the nucleus and the cytoplasm were unclear, and the arrangement was irregular. YSDZ and diazepam could alleviate the above pathological morphology (Figure 5).

### Network pharmacology analysis

**FIGURE 6:**
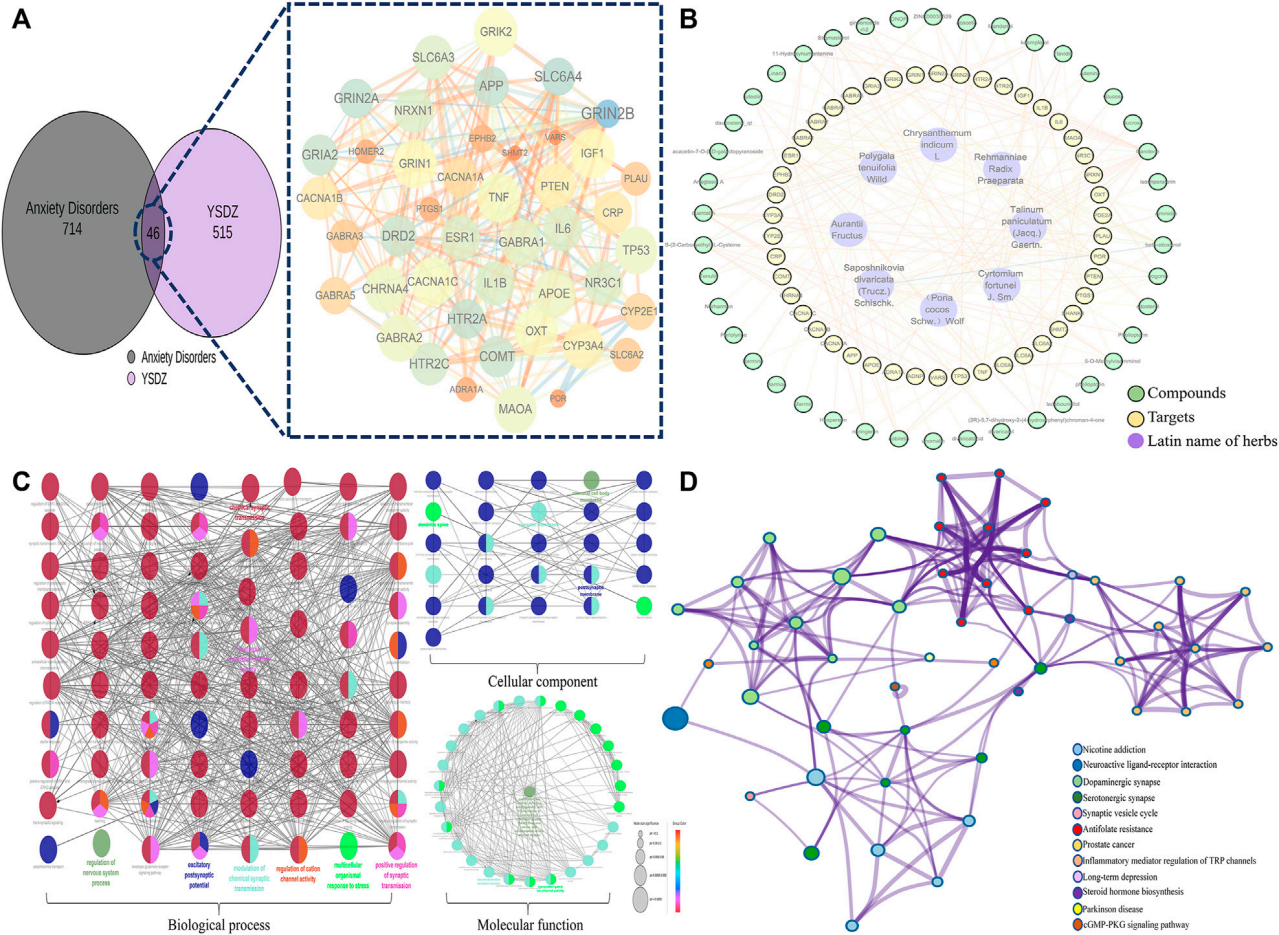
Comprehensive analysis of potential targets; **(A) **Potential target screening; **(B) **Compounds-Targets-Latin name of herbs network. **(C)** GO enrichment analysis; **(D)** KEGG enrichment analysis.

We identified a total of 515 non-repetitive targets and 41 active compounds pertaining to YSDZ based on the TCMSP and BATMAN-TCM databases. A total of 714 non-repetitive targets for anxiety disorders were obtained from Genecard, OMIM and Drugbank. After the intersection of YSDZ and anxiety disorder targets, we identified 46 potential therapeutic targets. Potential targets were imported into the STRING database and Cytoscape software, and the results were visualized (Figure 6A). The Latin names of active compounds and potential targets were imported into the Cytoscape software to visualize the anti-anxiety mechanism of YSDZ through the NetworkAnalyzer function (Figure 6B). The top 5 active compounds in degree value were Carotene, β-sitosterol, quercetin, Stigmasterol and kaempferol (Supplementary Table S1). The top 5 targets were Prostaglandin G/H synthase 1 (PTGS1), Gamma-aminobutyric acid receptor subunit alpha-1 (GABRA1), Tumor necrosis factor (TNF), Estrogen receptor (ESR1) and Interleukin-1 beta (IL1β) (Supplementary Table S2).

After importing the 46 potential therapeutic targets into the Metascape database, results of BP, MF, CC and KEGG enrichment analysis were generated. BP of targets mainly involved trans-synaptic signaling, modulation of chemical synaptic transmission, regulation of neurotransmitter levels, and regulation of synaptic transmission. MF mainly involved the neurotransmitter receptor activity, monoamine transmembrane transporter activity, and signaling receptor activator activity. CC mainly involved the synaptic membrane, presynaptic membrane, and axons. The results of KEGG enrichment analysis mainly involved the dopaminergic synapse, serotonergic synapse, and neuroactive ligand-receptor interaction (Figures 6C,D).

### Molecular docking and molecular dynamics simulation

**FIGURE 7:**
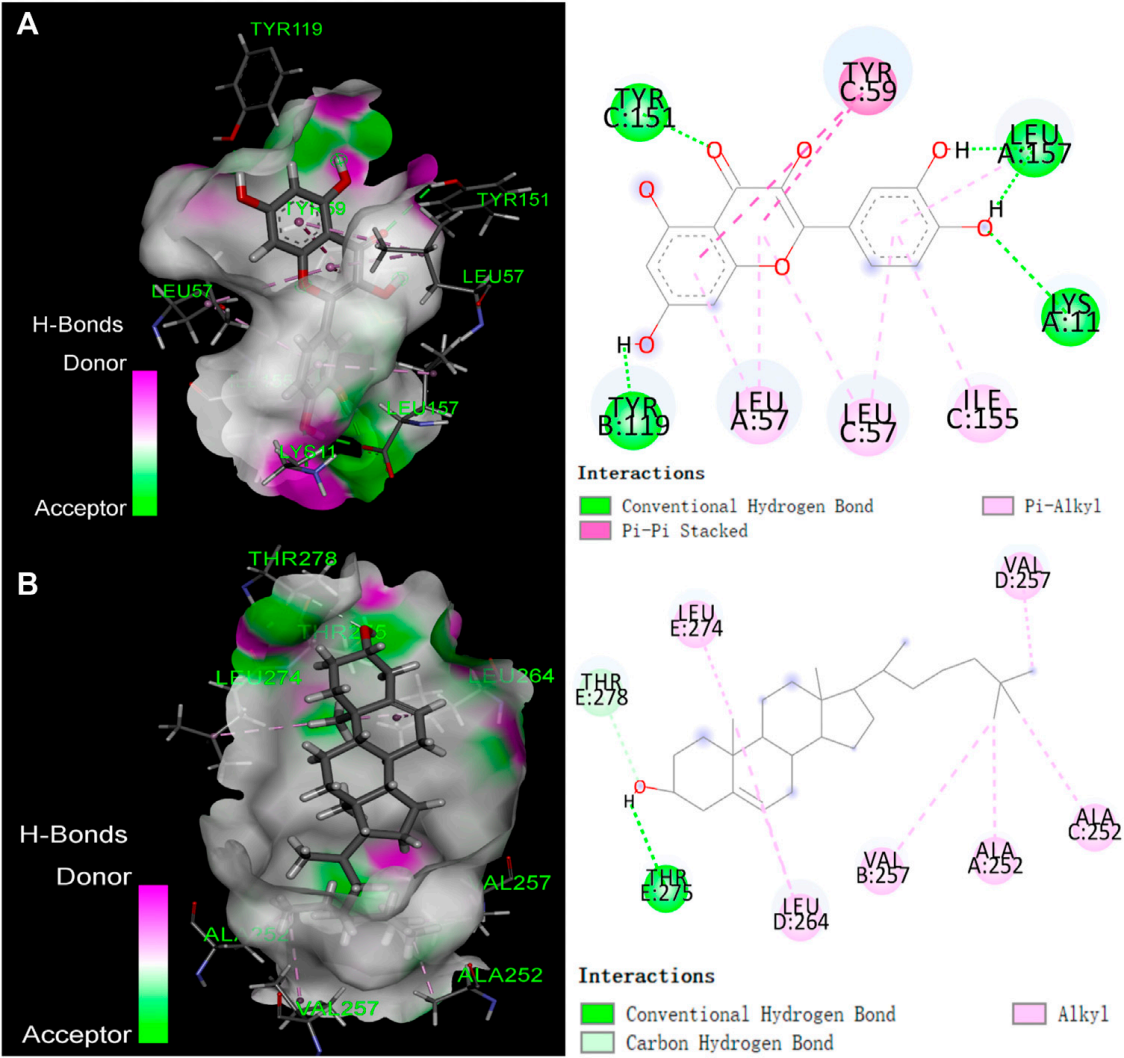
The binding mode of compounds with target proteins. **(A)** Quercetin-TNF; **(B)** β-Sitosterol-GABRA1.

The top 5 active compounds were simulated with the top 5 potential targets IL1B (PDB ID: 5R86; Resolution:1.5 Å), GABRA1 (PDB ID: 6 × 3T; Resolution:2.55 Å), PTGS1 (PDB ID: 3LN0; Resolution:2.2 Å), ESR1 (PDB ID: 7KCD; Resolution:1.80 Å), TNF (PDB ID: 7KPA; Resolution:2.30 Å). Ligand pertains the compound, while the receptor was the target protein and the affinity results are visualized. Lower affinity indicate more stable interactions, whereas we considered that a affinity lower than −4.25 kcal/mol was a cutoff indicative of a possible interaction (Dan et al., 2020). Results showed (Supplementary Table S3) that all binding energies were less than −5 kcal/mol, and the average binding energy was −7.7764 kcal/mol, indicating that the five active compounds were possilby binded to the five potential core targets. The compound-protein interaction was visualized using Pymol 2.1 software (the compound with the lowest binding energy was selected for each target), and the binding mode of the compound and the protein was obtained (Figure 7).

**FIGURE 8:**
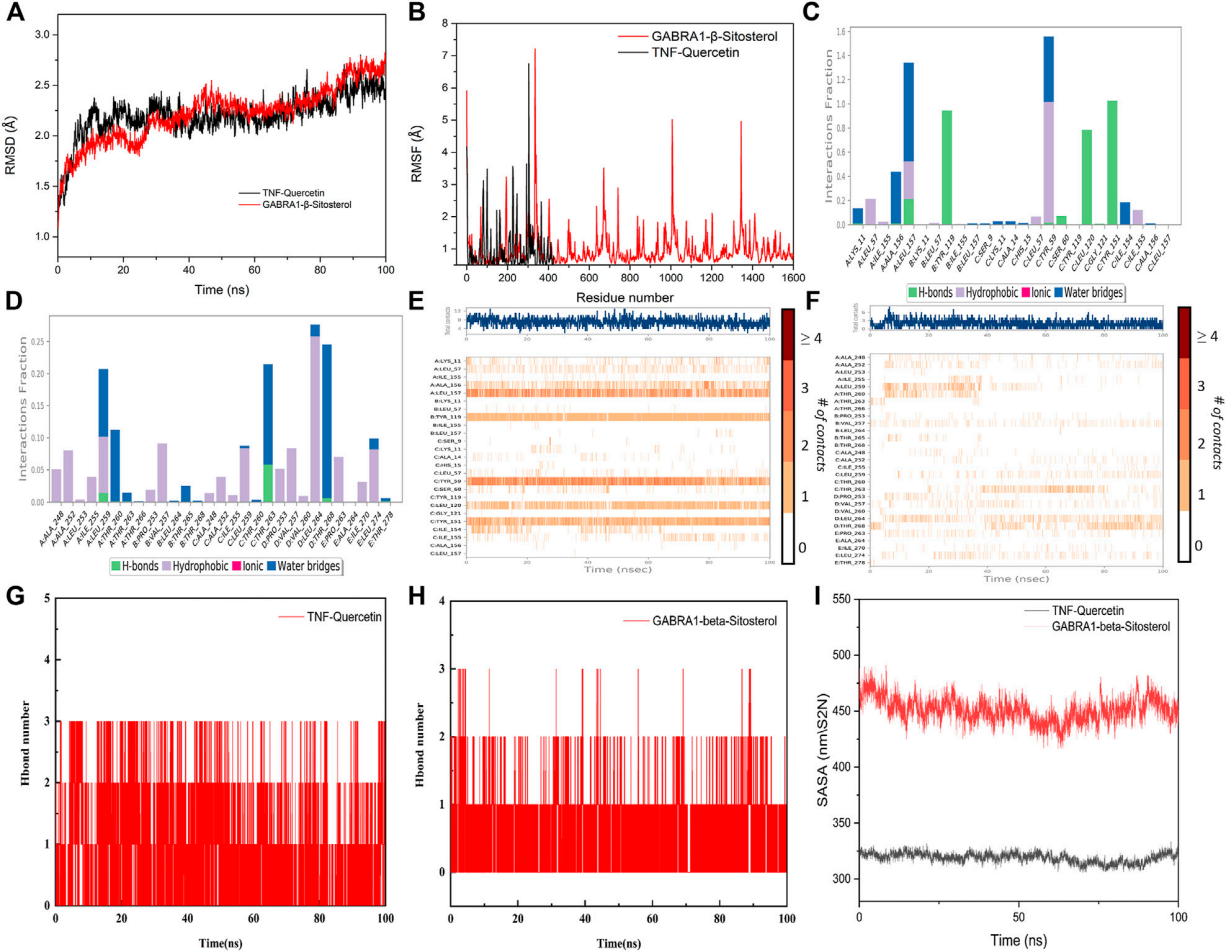
Molecular dynamics simulation of active compounds of YSDZ and key target proteins. **(A)** RMSD values during molecular dynamics simulations. **(B)** RMSF values during molecular dynamics simulations. **(C–F)** Compound-target interaction residues. **(C,E)** is TNF-Quercetin. **(D,F)** is GABRA1-β-Sitosterol. **(G,H)** Hydrogen Bond Analysis. **(I)** SASA area analysis.

Taking into account the results of network pharmacology and molecular docking, TNF and GABRA1 were selected for molecular dynamics simulation to further study the interaction between the active compounds of YSDZ and the target proteins. Monitoring the RMSD of a protein can provide insight into its structural conformation and indicate whether the simulation is in equilibrium. Results showed that the RMSDs of the two groups of complexes were less than 2.5 Å, while the complexes reached dynamic equilibrium in a relatively short period, indicating that small molecules formed stable complexes with proteins (Figure 8A). In parallel, RMSF reflects the conformational changes of each amino acid within the protein. In the TNF-Quercetin (Binding free energy: −85.42 ± 4.035 kJ/mol) model it was found that some amino acids in TNF changed greatly, which was mainly due to the breaking of the amino acid chain in this part of the protein. Other parts of the proteins suffered small conformational changes, reflecting the stability of this complex. With regards to the GABRA1-β-Sitosterol (Binding free energy: −66.716 ± 3.762 kJ/mol) combination, a small portion of amino acids (i.e., 330–345 and 1620−1635) suffered large conformational changes. These amino acids are mainly located in the loop region, which has little influence on the stability of the complex. Indeed, the binding site of β-Sitosterol was distant to the loop region of GABRA1 (Figure 8B).

For the TNF-Quercetin complex, it was found that Quercetin had strong hydrogen bond interactions with LEU-157, TYR-119, LEU-120, and TYR-151 that occurred early in the simulation process, suggesting that these hydrogen bonds are critical to stabilize Quercetin in the protein pocket. Additionally, the Quercetin showed strong hydrophobic interactions with LEU-57, TYR-59, and LEU-157 that may also stabilize the complex. In the GABRA1-β-Sitosterol complex, β-Sitosterol was found to be associated with hydrophobic amino acids ALA-248, ALA-252, ILE-255, LEU-259, VAL-257, PRO-253, LEU-264, PRO-263, and LEU- 274. In addition, the compound could also form hydrogen bonds with THR-263, which contributes to the stability of the complex. Therefore, the binding of β-Sitosterol to GABRA1 is primarily driven by hydrophobic interactions (Figures 8C–F).

In order to describe the hydrogen bonds between small molecules and proteins in more detail during the entire simulation process, we counted the hydrogen bonds between small molecules and protein pockets within 100 ns. The results show that β-Sitosterol and Quercetin have hydrogen bond interactions with proteins during the entire simulation process, and the number of hydrogen bonds is greater than or equal to 1, which plays an important role in stabilizing small molecules. It also shows that small molecules can form stable complexes with proteins (Figures 8G,H). In addition, according to the Solvent-accessible surface area (SASA), the SASA fluctuation range of the protein in the whole simulation process is small, which also indicates that the complex between the protein and the small molecule is relatively stable. Moreover, we found that SASA has a slight downward trend after 20 ns, which indicates that during the movement of the aqueous solution, the small molecule also improves the protein stability after the protein pocket is continuously adjusted to the optimal binding state (Figure 8I). In summary, it shows that small molecules bind well to proteins, and the complexes have high stability.

### Yangshendingzhi granules regulates expression of tumor necrosis factor-α and gamma-aminobutyric acid receptor subunit alpha-1

**FIGURE 9:**
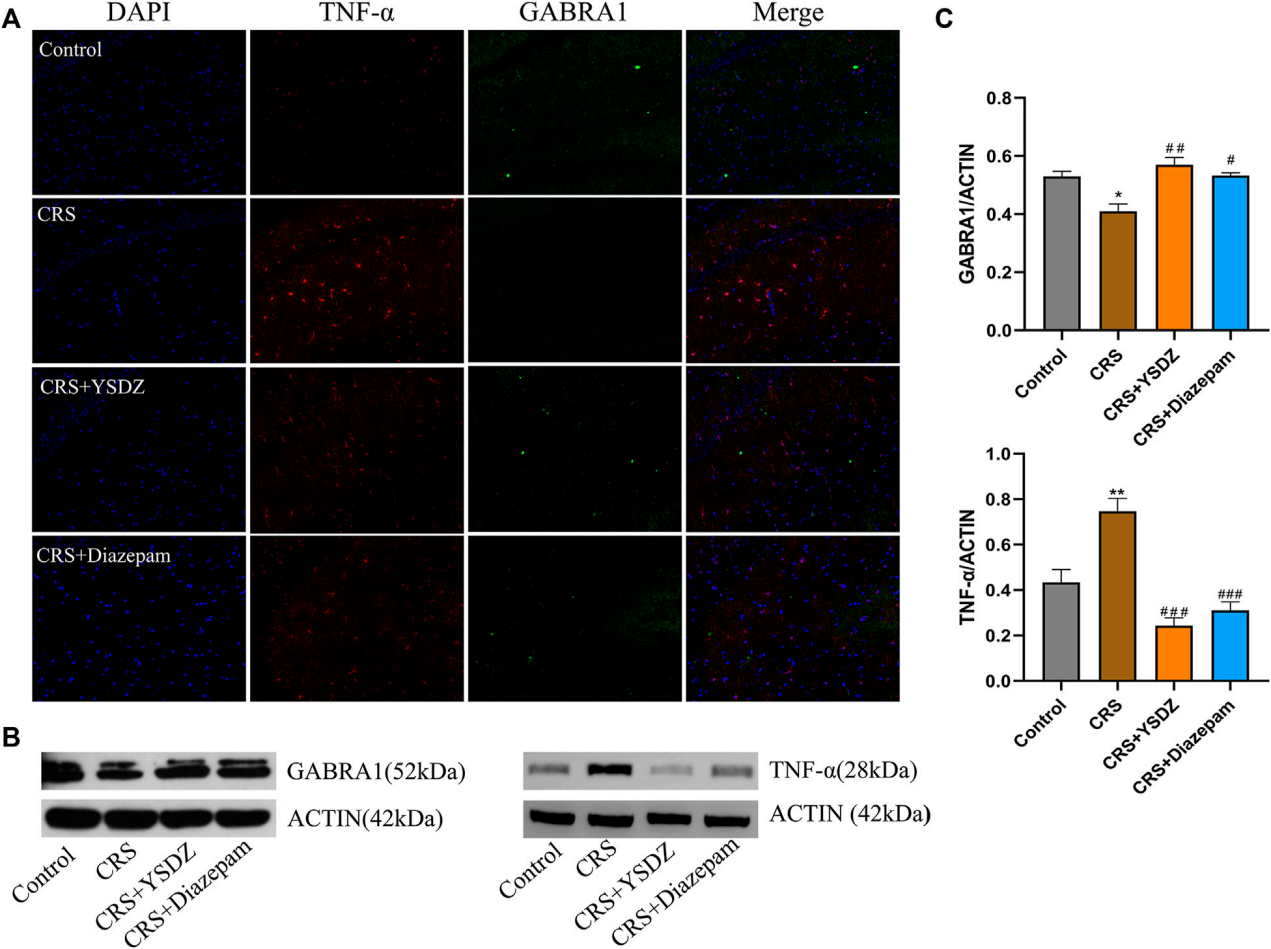
Verify protein expression. **(A)** Representative images of immunofluorescence staining of hippocampal slices for TNF-α (red) and GABRA1 (green); **(B)** Representative blots; **(C)** The protein expression levels of TNF-α and GABRA1 in WB. *p < 0.05, **p < 0.01 compared with Control; #*p* < 0.05, ##*p* < 0.01, ###*p* < 0.001 compared with CRS *via* one-way ANOVA.

Based on network pharmacology, molecular docking and MD results, the two targets with the best average affinity (GABRA1, TNF-α) were further analyzed. Combining the immunofluorescence and WB results, it was clear that when compared with control rats, CRS animals showed a decreased expression of GABRA1, which was reversed in YSDZ and diazepam rats. At the same time, compared with the control group, CRS caused the overexpression of TNF-α—an effect downregulated by YSDZ and diazepam (Figure 9).

### Yangshendingzhi granules improves hippocampal metabolic disorders

**FIGURE 10:**
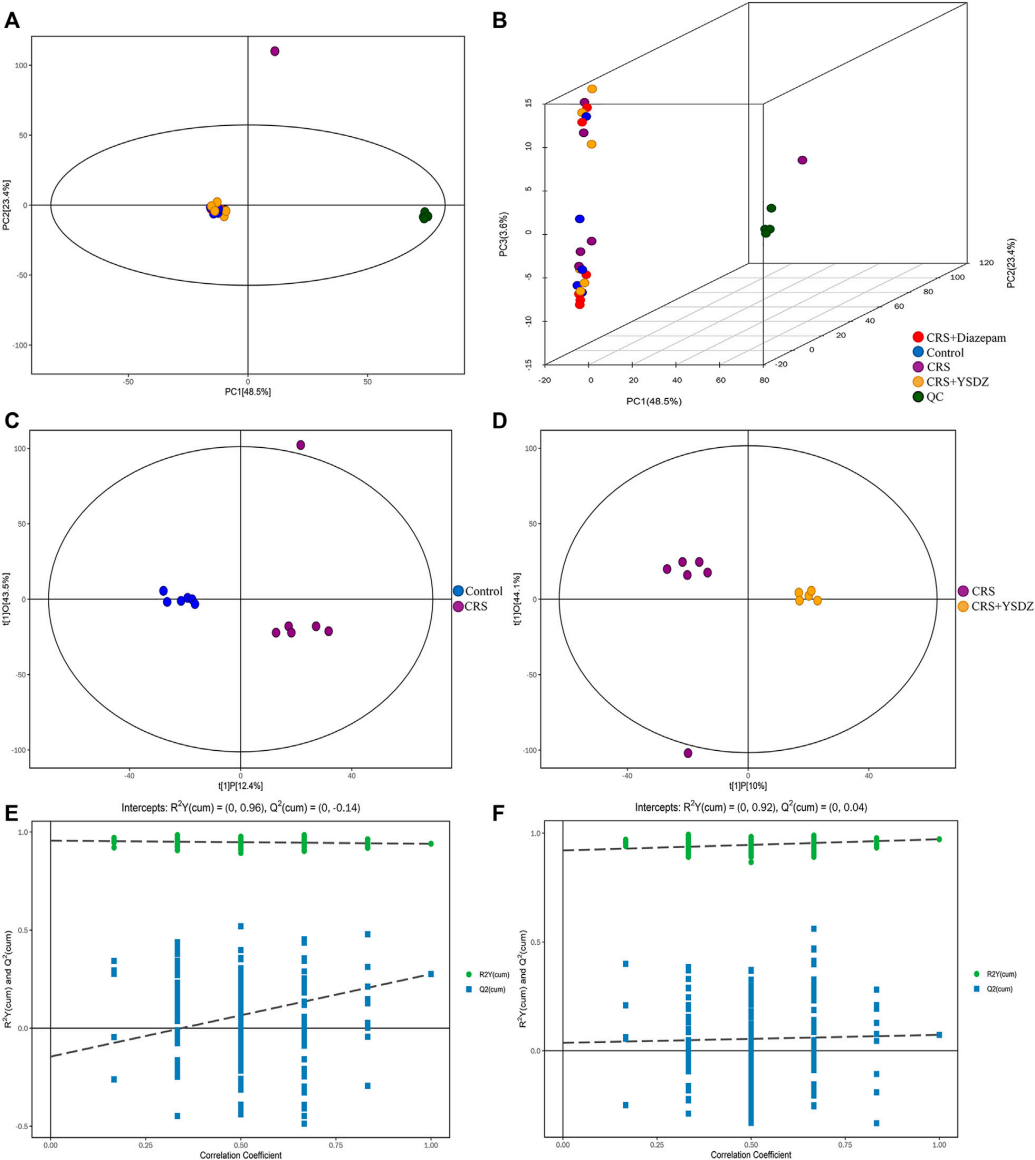
Multivariate statistical analysis of metabolites. **(A)** Score scatter plot of the PCA model; **(B)** 3D Score scatter plot of the PCA model. QC (quality control), *n* = 6 per group. **(C,D)** are score scatter plot of the OPLS-DA model.**(C)** is CRS vs. Control. **(D) **is CRS + YSDZ vs. CRS. **(E,F)** are permutation plot test of the OPLS-DA model. **(E) **is CRS vs. Control. **(F) **is CRS + YSDZ vs. CRS.

As an unsupervised model, PCA is useful to analyze the internal structure of the data, providing a relatively low-dimensional matrix (two- or three-dimensional). The effective use of a small number of principal components reduces the dimension of the dataset, thereby effectively highlighting the trend for the overall distribution of the metabolomics data and the differences between groups. It is evident from the PCA subplot that the sample was within the 95% confidence interval (Hotelling’s T-squared ellipse) (Figures 10A,B). The OPLS-DA method was used to identify metabolites differentially expressed between control and model groups, and between model and YSDZ groups. Metabolites were strikingly different within the abovementioned comparisons. The permutation test (number of times *n* = 200) was used to establish the corresponding OPLS-DA model to obtain the values of *R2* and Q2 values from the random model. Results showed that the two groups of models were robust and that there was no over fitting (Figures 10C–F). Based on the results of these tests, that measurements and findings were proved to be reliable.

**FIGURE 11:**
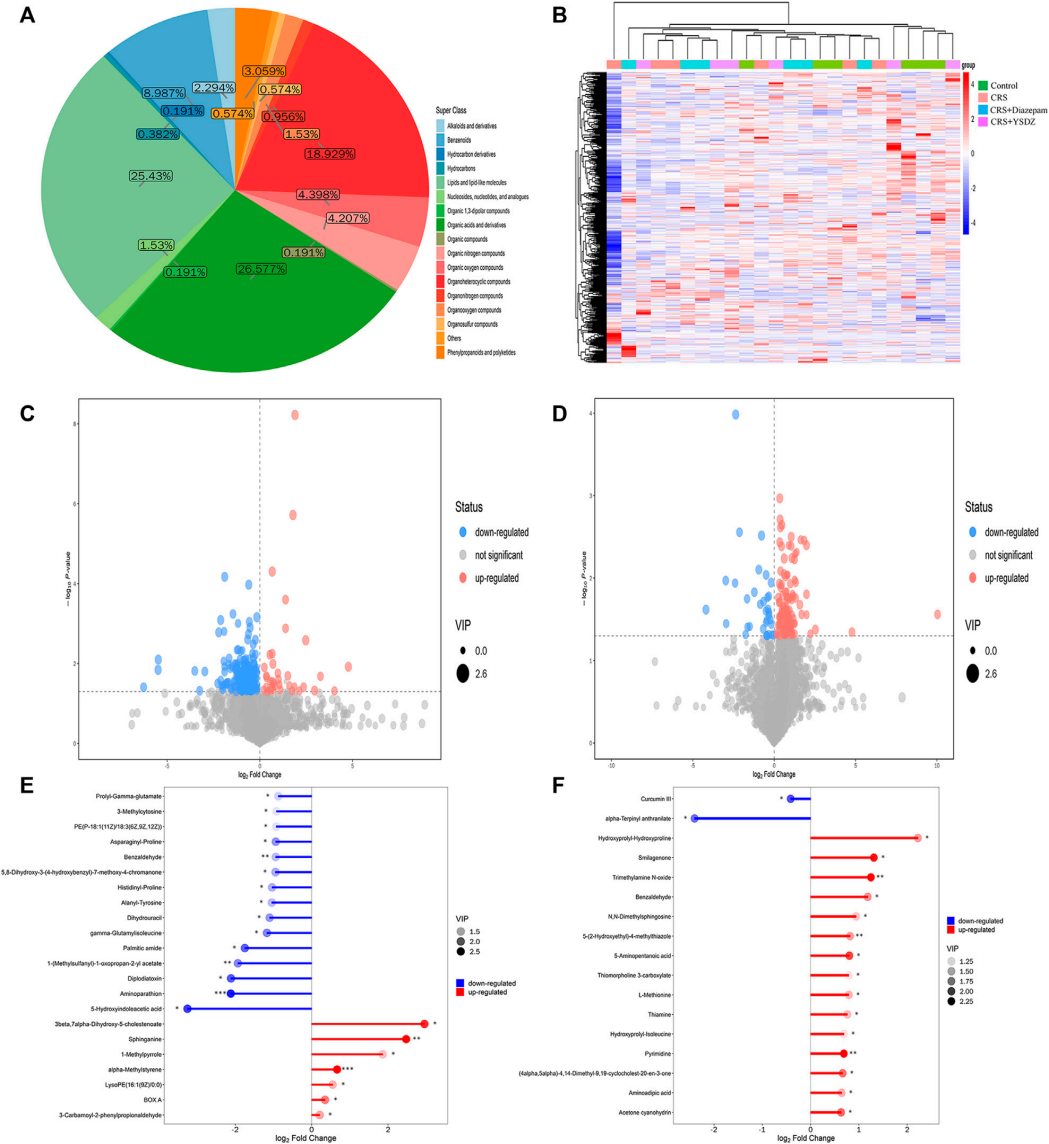
Metabolite comprehensive analysis.**(A) **Pie plot of metabolite classification and proportion. **(B) **Heatmap of hierarchical clustering analysis for all groups. The abscissa in the figure represents different sample groups, the ordinate represents all metabolites, the color blocks at different positions represent the relative expression levels of the metabolites at the corresponding positions, red indicates high expression of the substance, and blue indicates low expression of the substance. **(C,D)** are Volcano plot. The *X*-axis represents multiple changes of all substances compared in this group (logarithmic base 2), the *Y*-axis represents the *p*-value of student’ *t* test, and the size of the scatter point represents VIP values of the OPLS-DA model. Significantly upregulated metabolites are represented in red, while those significantly downregulated are represented in blue, unchanged metabolites are shown in gray. **(C)** is CRS vs. Control. **(D)** is CRS + YSDZ vs. CRS. **(E,F)** are Matchstick analysis. The first 15 metabolites most up or downregulated are displayed. The *X*-axis shows change after logarithmic transformation, the color depth of the point represents the VIP value, and the *Y*-axis represents differential metabolites. **(E)** is CRS vs. Control. **(F)** is CRS + YSDZ vs. CRS.

The identified metabolites were classified and counted according to the chemical classification information. The results showed that the most abundant metabolites identified were organic acids and derivatives, followed by organic 1,3-dipolar compounds, organoheterocyclic compounds, etc (Figure 11A). All identified metabolites were subjected to cluster heatmap analysis, and cluster heatmaps were drawn to visualize the distribution of metabolites among groups and their relationships (Figure 11B). After initial checks regarding the validity of measurements, differential metabolites with a VIP > 1 and *p* < 0.05 were identified. We found 49 metabolites differentially expressed in CRS rats when compared to controls. A total of 42 metabolites were downregulated in CRS rats, including 5-Hydroxyindoleacetic acid, Aminoparathion and Diplodiatoxin. Parallelly, the levels of 7 metabolites, including 3-beta, 7-alpha-Dihydroxy-5-cholestenoate and Sphinganine were increased in CRS rats when compared to those of control ones. Compared with the CRS group, the intervention of YSDZ changed the levels of 27 metabolites. Metabolites such as Hydroxyprolyl-Hydroxyproline, Smilagenone, and Trimethylamine N-oxide increased after intervention of YSDZ, while alpha-Terpinyl anthranilate and Curcumin III were down-regulated (Figures 11C–F). In addition, metabolites such as Pyrimidine, Thiamine, 5-(2-Hydroxyethyl)-4-methylthiazole, 3-Formyl-6-hydroxyindole, Acetone cyanohydrin, 1-Methyladenine, Benzaldehyde, 5-Aminopentanal,Dimethylglycine, 2-(Methylamino) benzoic acid, Aminoadipic acid and Toluene were down-regulated in CRS rats and upregulated in YSDZ rats (Supplementary Table S4).

**FIGURE 12:**
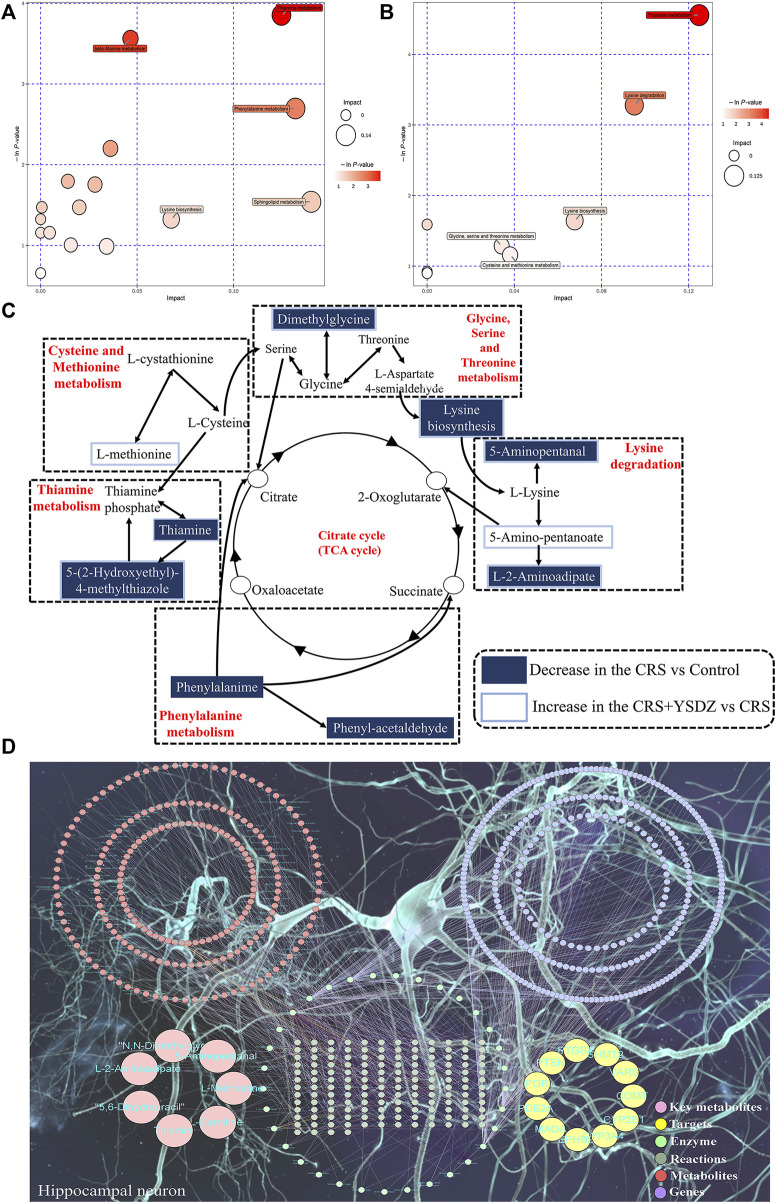
Comprehensive analysis of metabolic pathways. **(A,B)** are Pathway analysis. Each bubble in the bubble map represents a metabolic pathway, where the *Y*-axis and bubble size represent the impact factor of this pathway in the topology analysis, and the *X*-axis and bubble color represent the *p*-value of enrichment analysis. **(A)** is CRS vs. Control. **(B)** is CRS + YSDZ vs. CRS. **(C) **Representative metabolic pathways found in metabolomics analysis. **(D) **Key metabolite-Target-Enzyme-Reaction-Compound-Gene networks.

A metabolic pathway analysis of differential metabolites was performed using the KEGG (http://www.kegg.com) (Kanehisa et al., 2000)and the MetaboAnalyst databases (Xia et al., 2015). Results indicated that the anti-anxiety effect of YSDZ was related to thiamine metabolism, cystine and methionine metabolism, Lysine biosynthesis, Lysine degradation and other pathways (Figures 12A–C). At the same time, the differential metabolites and potential therapeutic targets identified through network pharmacology were imported into the MetScape of Cytoscape, and the network diagram of differential metabolites-potential therapeutic. Differentially expressed metabolites were visualized with regards to their potential therapeutic targets, and regulatory relationships. Altogether, these data reflect multiple mechanisms through which several components of YSDZ exert their anxiolytic effect (Figure 12D).

## Discussion

TCMs are often composed of several components with different mechanisms of action. In this regard, network pharmacology is a promising method to explore and elucidate the complex mechanism of TCMs (Pan et al., 2020). Therefore, we used network pharmacology to predict that the active ingredients of YSDZ, such as carotene, beta-sitosterol, quercetin, stigmasterol, and kaempferol act on potential targets such as GABRA1, TNF, IL1β, ESR1, and PTGS1. GABA is widely distributed in the mammalian central nervous system and the main inhibitory neurotransmitter in the mammalian brain (Miller et al., 2019), which is closely related to the pathogenesis of anxiety (Kalueff et al., 2017). GABA receptors, also known as γ-aminobutyric acid type A receptors (such as GABRA1), are a class of ligand-gated ion channels (Kanaumi et al., 2006). It is a class of ligand-gated ion channel proteins with a 5-transmembrane structure and belongs to the cysteine ring family (Zhu et al., 2018). Drugs regulate GABA-mediated postsynaptic membrane hyperpolarization by acting on GABAAR, which is of great significance in regulating brain memory, consciousness and sleep. There is evidence showing that GABRA1 can improve anxiety behavior in rats (Gupta et al., 2019). In parallel, the inflammation is also closely related to the pathophysiology of depression and anxiety (Kiecolt-Glaser et al., 2011). TNF-α and IL-1β are pro-inflammatory cytokines that affect neuronal function by regulating oxidative stress, apoptosis, and metabolism, and impairing neuronal branching processes (Wang et al., 2019) There is evidence demonstrating that TNF-α plays a critical role in the cognitive decline and the progression of depression and anxiety, while both IL-1β and TNF-α are elevated in patients with depression. Interestingly, blocking TNF-α signaling improves anxiety and depressive-like behaviors (Alshammari et al., 2020). Another target that has been identified was the estrogen receptor ESR1. The cellular effects of estrogen are mediated by two estrogen receptors (ERα, ERβ), which regulate the physiological processes such as cell growth and development, and closely related to the pathological processes such as inflammation, osteoporosis, cancer, and neurodegeneration (Jia et al., 2015). ERα has significant effects on mood and cognition, and variants in estrogen receptors may alter estrogen signaling and increase susceptibility to depression, anxiety in women (Gutiérrez-Muñoz et al., 2018). Previous evidences showed that specific ESR1 polymorphisms might contribute to the increased susceptibility to the development of anxiety disorders (Borrow et al., 2017). Through network pharmacology analysis, it was predicted that YSDZ could act on the above-mentioned targets to play a role in intervening anxiety.

Quercetin is a flavonoid that was widely present in vegetables and fruits with the anti-inflammatory, antioxidant, antiviral, and anticancer effects (Li et al., 2016). Previous evidences have proved that quercetin could improve the neuroinflammation caused by the lipopolysaccharide by inhibiting the production of pro-inflammatory factors such as TNF-α and IL-1β, thereby exerting anxiolytic effects (Lee et al., 2020). Our results of molecular docking showed that the binding energy of quercetin to TNF-α was −9.84 kcal/mol and quercetin formed hydrogen bonds with the residues of TYR-151, TYR-59, LYS-11, LEU-57, TYR-119, and LEU-157. Quercetin was able to bind to the active pocket of TNF-α, and the stability of the TNF-α-quercetin complex was further verified by MD. Meanwhile, the flavonoid kaempferol, which is the main component of various fruits and vegetables, was shown to alleviate the oxidative stress, inflammatory responses (Gao et al., 2019) and anxiety (Silva et al., 2021). In molecular docking, the binding modes of kaempferol and quercetin were very similar since they were shown to form the stable hydrogen bonds and hydrophobic interactions with the active site of the target protein.

The phytosterols belong to a class of steroidal compounds with natural biological activities that are relevant to the metabolism of plant cells. These compounds are abundant in the roots, stems, leaves, seeds, and fruits of various oil plants and TCMs. Interestingly, β-sitosterol is the most representative phytosterol found in TCMs (Huang et al., 2019). As previously reported,β-sitosterol had a anxiolytic effect (Panayotis et al., 2021). Meanwhile, stigmasterol is a natural product that is present in various medicinal plants (Morgan et al., 2021). Previous studies have found that stigmasterol exerts anxiolytic effects by positively regulating GABA receptors. Thus, stigmasterol has been used as a candidate steroid drug for the treatment of neurological diseases (Karim et al., 2021). The final compound identified was carotene, which is the main source of vitamin A, presenting in three forms: *α*, *ß* or *γ* carotene, among which β-carotene has been widely studied. In fact, β-carotene intake has been reported to be inversely associated with the anxiety in early perimenopausal United States women (Li et al., 2021).

To further verify the anxiolytic effect of YSDZ, anxiety was induced in rats through CRS. Results showed that CRS rats had reduced open-field, light-dark box movement distance, OT% and OE% and other anxiety-like behaviors. CRS Rats after YSDZ intervention show improved anxiety-like behaviors. Moreover, YSDZ also reversed TNF-αoverexpression and improved the downregulation of GABRA1 expression caused by CRS. Based on these results, the anxiolytic effects of YSDZ and part of the predicted results from initial network pharmacology predictions were comfirmed. The hippocampus is one of the areas where the neural stem cells of the brain are located, which can maintain the generation of new neurons. The neurogenesis of the hippocampus is related to learning, memory and emotion regulation, and its damage is closely related to the occurrence of psychological disorders such as anxiety and depression (Jung et al., 2020). The results of this study showed that after CRS, the number of hippocampal CA1 neurons was slightly reduced, the neurons were atrophic and stained, the cell body was atrophic and deformed, the boundary between nucleus and cytoplasm was unclear, and the arrangement was irregular. But YSDZ could alleviate neuropathic injury in hippocampus, which was similar to previous reports (Yan et al., 2021).

In hippocampal metabolomics experiments, it was found that the anti-anxiety mechanism of YSDZ was closely related to thiamine metabolism, lysine biosynthesis, lysine degradation and cysteine and methionine metabolism. YSDZ reversed the downregulation of thiamine induced by CRS. Of note, thiamine is a water-soluble vitamin that plays an important role in the biological processes such as glucose metabolism (Polegato et al., 2019). There is evidence showing that the deficiency of the thiamine led to long-term neurobehavioral deficits, such as anxiety disorders (Li et al., 2020). In fact, thiamine supplementation can prevent the occurrence of anxietious behaviors (Markova et al., 2017). At the same time, there is evidence showing that thiamine deficiency induced the activation of microglia in the thalamus (Bowyer et al., 2018), thus contributing to neuroinflammation (Su et al., 2020). Therefore, there might be a potential activation of pro-inflammatory cytokines such as TNF-α and IL-1βupon excessive thiamine deficiency. Finally, previous studies have demonstrated that excessive deficiency of thiamine could impair GABAergic neurons (de et al., 2020), and indirectly induce the onset of anxiety.

Lysine plays an important role in promoting the growth and development of human beings and enhancing their immunity. It also plays an important role in the treatment of anxiety. It has been reported in randomized clinical trials that the combination of L-lysine and L-arginine can effectively reduce the patients’ score of anxiety with no obvious side effects (Lakhan et al., 2010). There are also evidences showing that the increases in L-lysine nutrient load could reduce the stress-induced anxiety (Smriga et al., 2003). At the same time, we found that YSDZ up-regulated the metabolism of cysteine and methionine, which is in consistence with the result of a previous report showing that TCM could alleviate depression- and anxiety-like behaviors in rats, through the upregulation of cysteine and methionine metabolism (Qu et al., 2019).

## Conclusion

We found that the anxiolytic effect of YSDZ are associated with the regulation of GABRA1 and TNF-α, the amelioration of pathological damage in hippocampal CA1 region, and the upregulation of thiamine, cysteine, and methionine metabolites as well as lysine biosynthesis and degradation. It is preliminarily revealed that YSDZ exerts anxiolytic effects through the characteristics of multi-component, multi-target, and multi-pathway. However, there are several limitations in this study. The results of this study are limited to hippocampal metabolomics, therefore the metabolites in other regions of the brain and/or the serum remains unclear and need to be assessed in future. Finally, the active compounds and pathways predicted in network pharmacology are necessary to be further verified. Therefore, future research should focus on the monomeric compounds of YSDZ to clarify the abovementioned limitations.

## Data Availability

The original contributions presented in the study are included in the article/Supplementary Material, further inquiries can be directed to the corresponding authors.
