# Supplementary material for: Anxiolytic effect of YangshenDingzhi granules: Integrated network pharmacology and hippocampal metabolomics
Source: Front Pharmacol. 2022 Oct 31;13:966218. doi: 10.3389/fphar.2022.966218 (PMC9659911; doi:10.3389/fphar.2022.966218)
Supplement: Supplementary file 1 [file Table1.DOCX]

**Table 1** Information of potential active compounds of YSDZ.

| Molecule Name | Molecules structure | Molecular formula | PubChem CID | Database |
| --- | --- | --- | --- | --- |
| Carotene | 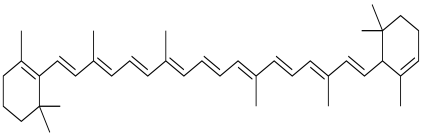 | [C](https://pubchem.ncbi.nlm.nih.gov/#query=C40H56)_[40](https://pubchem.ncbi.nlm.nih.gov/#query=C40H56)_[H](https://pubchem.ncbi.nlm.nih.gov/#query=C40H56)_[56](https://pubchem.ncbi.nlm.nih.gov/#query=C40H56)_ | 6419725 | BATMAN |
| β-sitosterol | 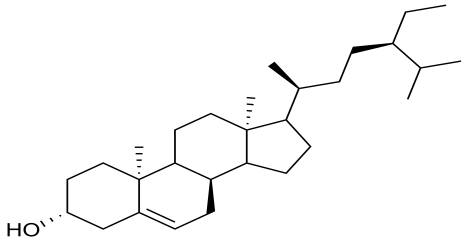 | [C](https://pubchem.ncbi.nlm.nih.gov/#query=C29H50O)_[29](https://pubchem.ncbi.nlm.nih.gov/#query=C29H50O)_[H](https://pubchem.ncbi.nlm.nih.gov/#query=C29H50O)_[50](https://pubchem.ncbi.nlm.nih.gov/#query=C29H50O)_[O](https://pubchem.ncbi.nlm.nih.gov/#query=C29H50O) | 521199 | TCMSP |
| Quercetin | 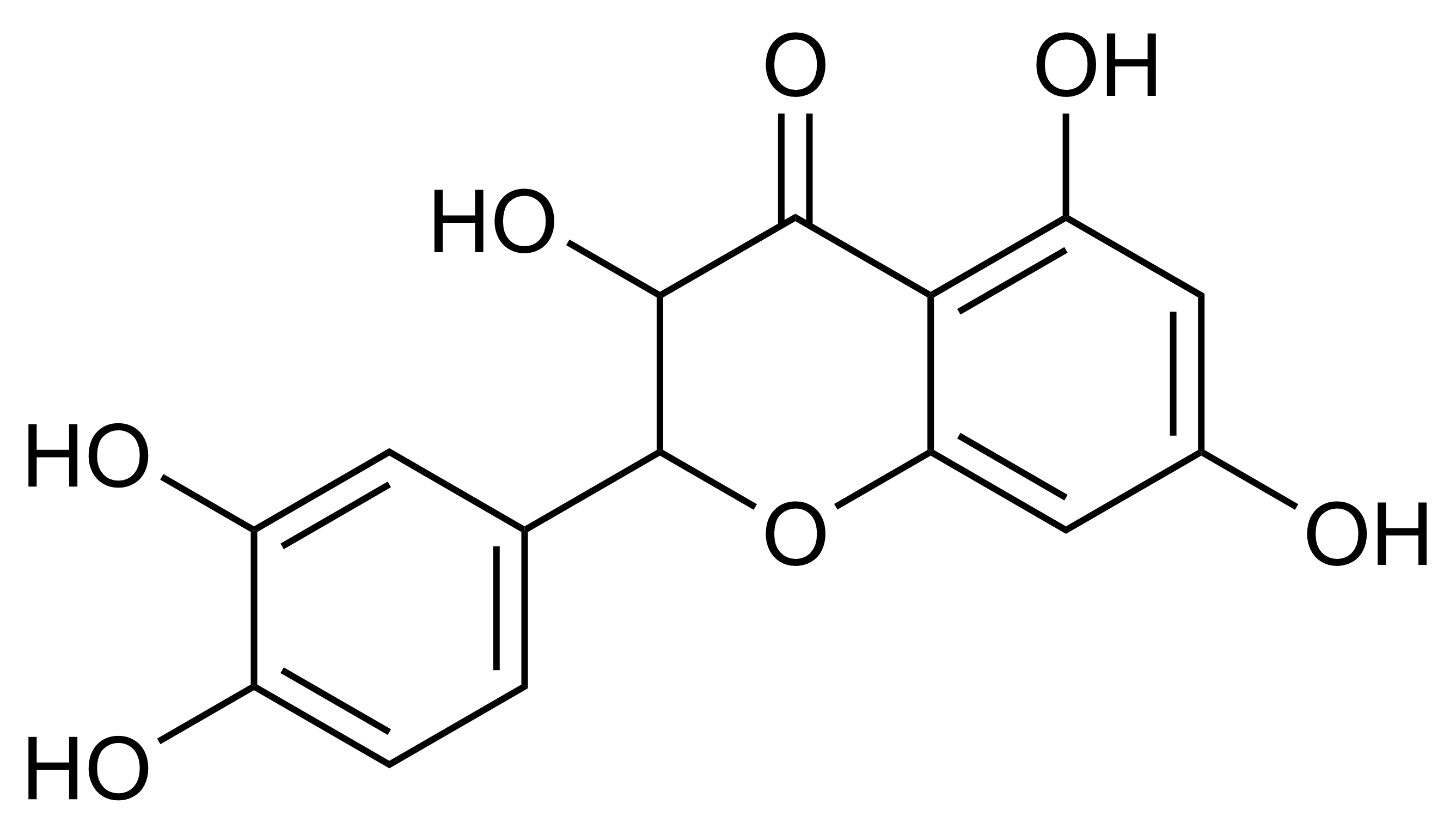 | [C](https://pubchem.ncbi.nlm.nih.gov/#query=C15H10O7)_[15](https://pubchem.ncbi.nlm.nih.gov/#query=C15H10O7)_[H](https://pubchem.ncbi.nlm.nih.gov/#query=C15H10O7)_[10](https://pubchem.ncbi.nlm.nih.gov/#query=C15H10O7)_[O](https://pubchem.ncbi.nlm.nih.gov/#query=C15H10O7)_[7](https://pubchem.ncbi.nlm.nih.gov/#query=C15H10O7)_ | 5280343 | TCMSP |
| Stigmasterol | 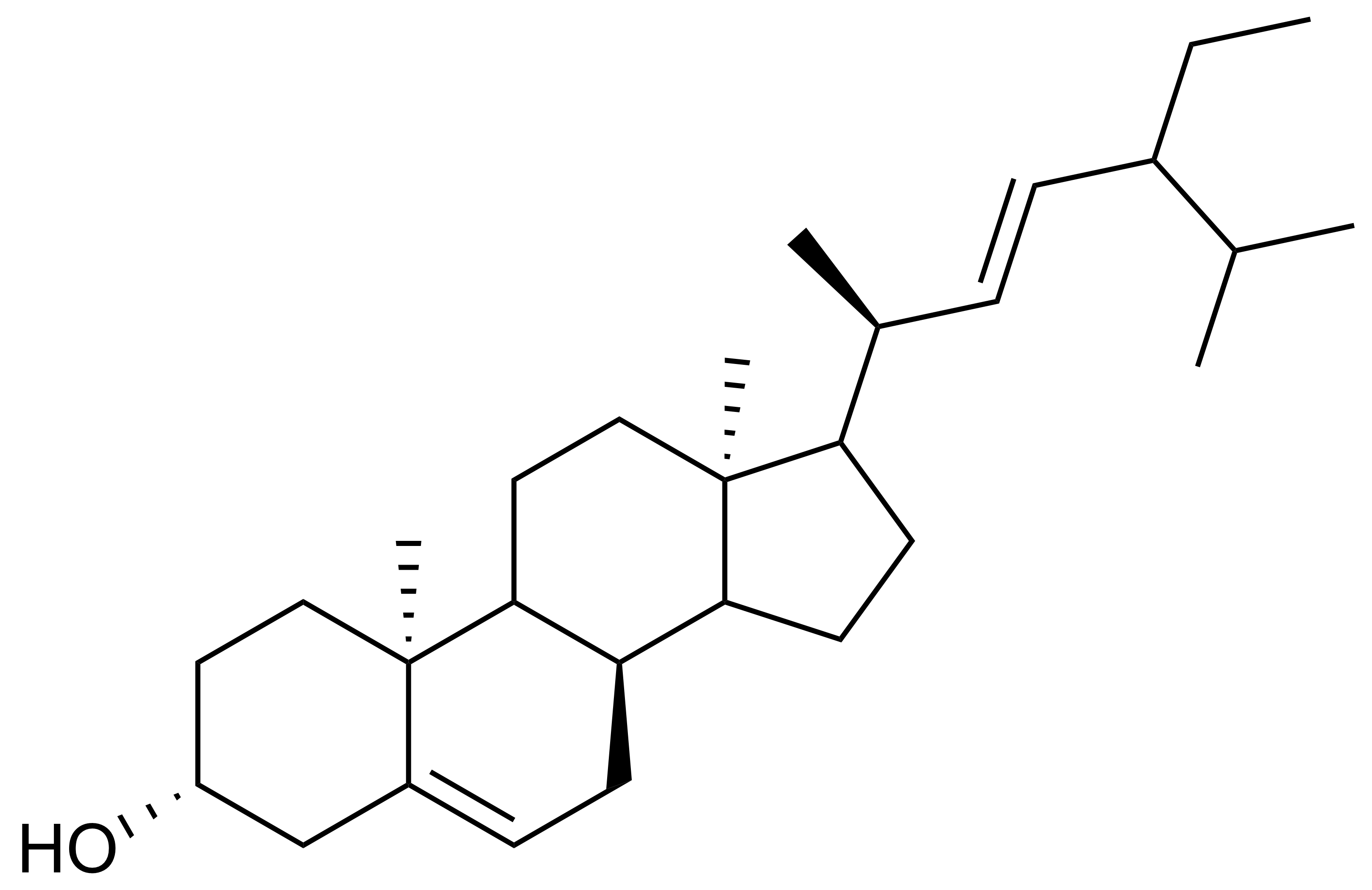 | [C](https://pubchem.ncbi.nlm.nih.gov/#query=C29H48O)_[29](https://pubchem.ncbi.nlm.nih.gov/#query=C29H48O)_[H](https://pubchem.ncbi.nlm.nih.gov/#query=C29H48O)_[48](https://pubchem.ncbi.nlm.nih.gov/#query=C29H48O)_[O](https://pubchem.ncbi.nlm.nih.gov/#query=C29H48O) | 5280794 | TCMSP |
| kaempferol | 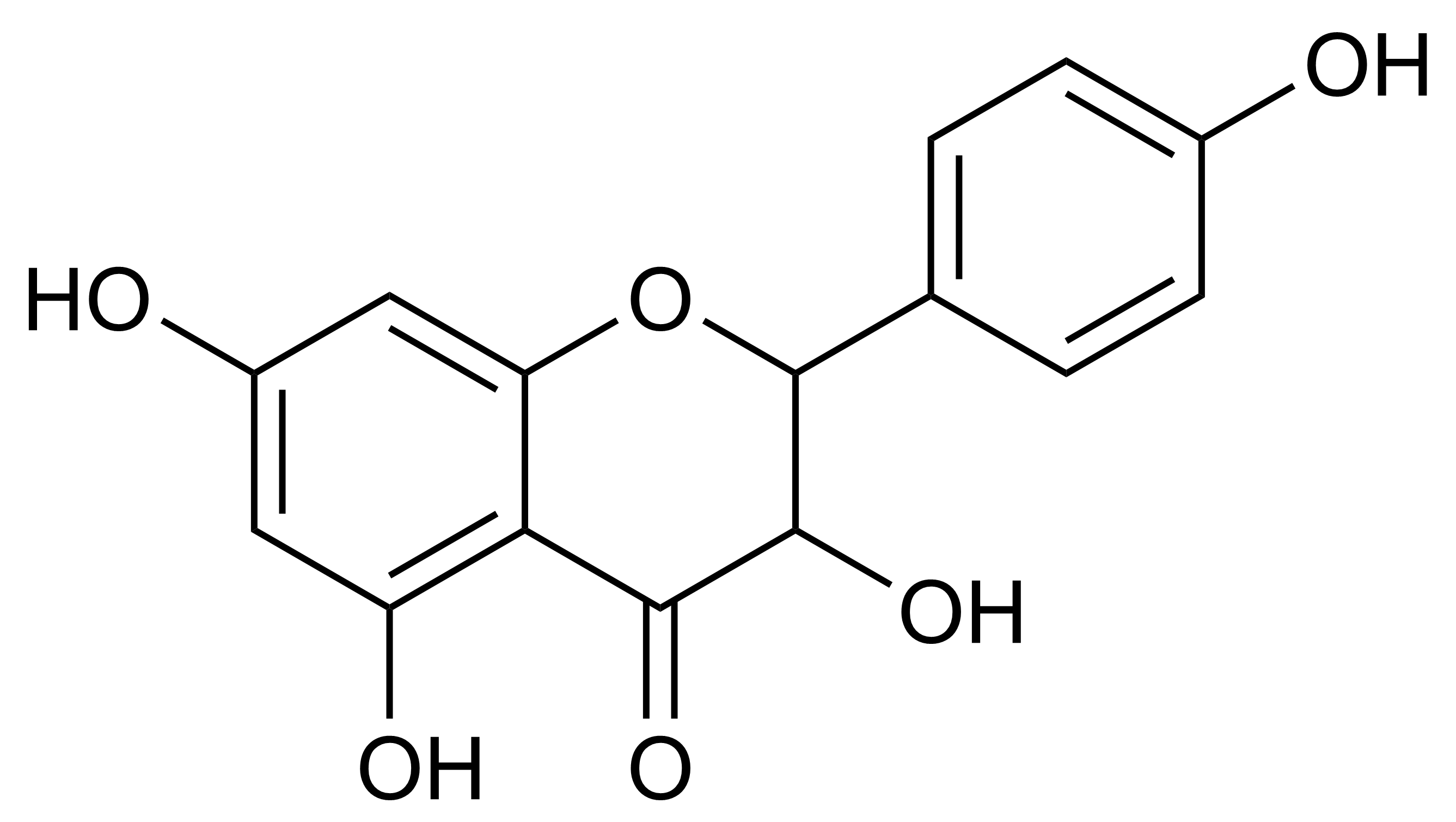 | [C](https://pubchem.ncbi.nlm.nih.gov/#query=C15H10O6)_[15](https://pubchem.ncbi.nlm.nih.gov/#query=C15H10O6)_[H](https://pubchem.ncbi.nlm.nih.gov/#query=C15H10O6)_[10](https://pubchem.ncbi.nlm.nih.gov/#query=C15H10O6)_[O](https://pubchem.ncbi.nlm.nih.gov/#query=C15H10O6)_[6](https://pubchem.ncbi.nlm.nih.gov/#query=C15H10O6)_ | 5280863 | TCMSP |
