# Supplementary material for: Anxiolytic effect of YangshenDingzhi granules: Integrated network pharmacology and hippocampal metabolomics
Source: Front Pharmacol. 2022 Oct 31;13:966218. doi: 10.3389/fphar.2022.966218 (PMC9659911; doi:10.3389/fphar.2022.966218)
Supplement: Supplementary file 2 [file Table2.DOCX]

**Table 2** Information of potential key anti-anxiety targets of YSDZ.

| Gene symbol | Degree | Uniprot ID | Protein name |
| --- | --- | --- | --- |
| PTGS1 | 16 | [P23219](https://www.uniprot.org/uniprot/P23219) | **Prostaglandin G/H synthase 1** |
| GABRA1 | 13 | [P14867](https://www.uniprot.org/uniprot/P14867) | **Gamma-aminobutyric acid receptor subunit alpha-1** |
| TNF | 8 | [P01375](https://www.uniprot.org/uniprot/P01375) | **Tumor necrosis factor** |
| ESR1 | 8 | [P03372](https://www.uniprot.org/uniprot/P03372) | **Estrogen receptor** |
| IL1β | 4 | [P01584](https://www.uniprot.org/uniprot/P01584) | **Interleukin-1 beta** |
