# Supplementary material for: Anxiolytic effect of YangshenDingzhi granules: Integrated network pharmacology and hippocampal metabolomics
Source: Front Pharmacol. 2022 Oct 31;13:966218. doi: 10.3389/fphar.2022.966218 (PMC9659911; doi:10.3389/fphar.2022.966218)
Supplement: Supplementary file 3 [file Table3.DOCX]

**Table 3** Docking results of core compounds and core targets.

| Proteins | PDB ID | Protein structure | Compounds | Affinity (kcal/mol) |
| --- | --- | --- | --- | --- |
| IL1β | 5R86 | 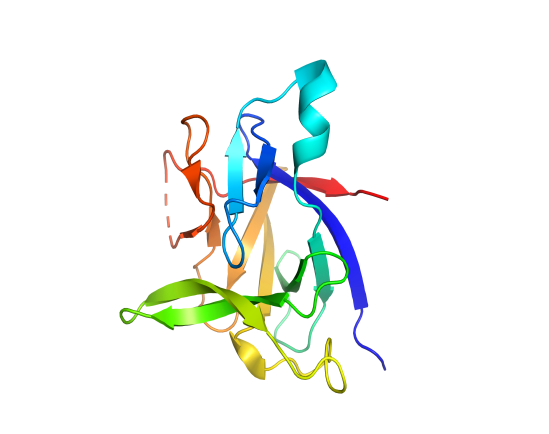 | Quercetin  Stigmasterol  Kaempferol  β-sitosterol  Carotene | -7.62  -6.84  -7.39  -7.03  -6.81 |
| GABRA1 | 6X3T | 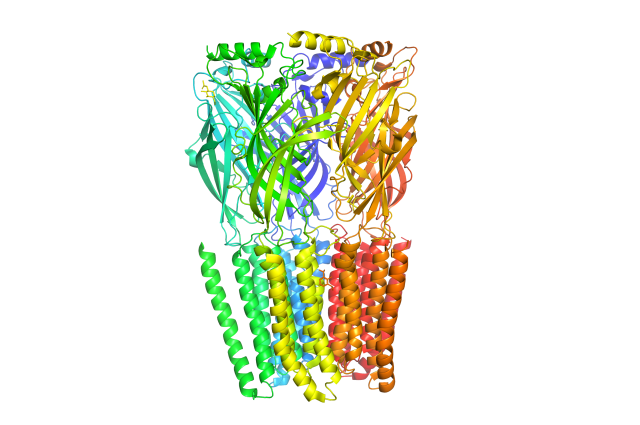 | Quercetin  Stigmasterol  Kaempferol  β-sitosterol  Carotene | -7.22  -7.46  -7.91  -8.11  -7.94 |
| PTGS1 | 3LN0 | 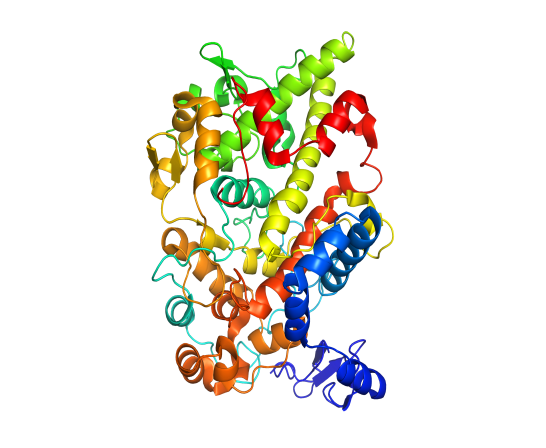 | Quercetin  Stigmasterol  Kaempferol  β-sitosterol  Carotene | -7.86  -7.02  -7.78  -7.14  -6.58 |
| ESR1 | 7KCD | 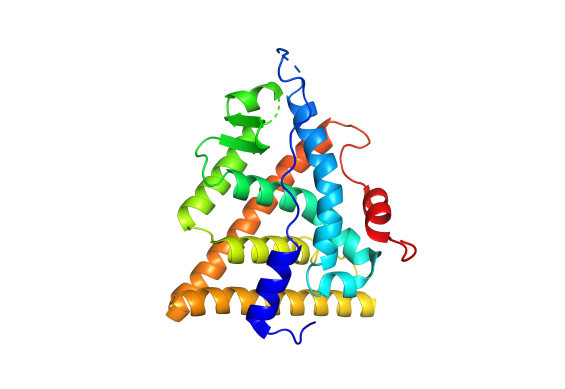 | Quercetin  Stigmasterol  Kaempferol  β-sitosterol  Carotene | -7.69  -6.83  -7.92  -7.06  -6.54 |
| TNF | 7KPA | 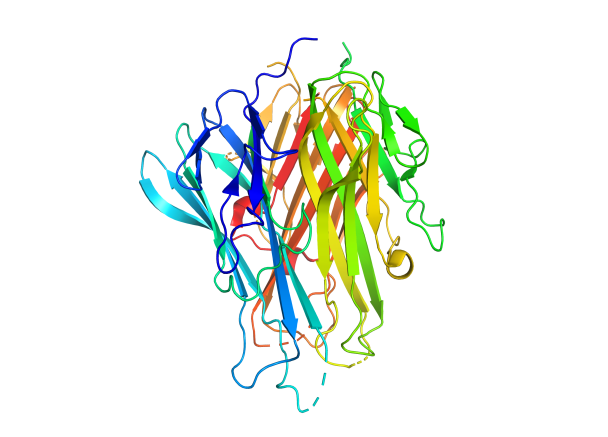 | Quercetin  Stigmasterol  Kaempferol  β-sitosterol  Carotene | -9.84  -9.25  -9.23  -9.68  -9.66 |
