# Supplementary material for: Anxiolytic effect of YangshenDingzhi granules: Integrated network pharmacology and hippocampal metabolomics
Source: Front Pharmacol. 2022 Oct 31;13:966218. doi: 10.3389/fphar.2022.966218 (PMC9659911; doi:10.3389/fphar.2022.966218)
Supplement: Supplementary file 4 [file Table4.DOCX]

Table 4 Differentially expressed metabolites between different groups.

| NO | Metabolites | CRS vs Control | | | | YSDZ vs CRS | | | |
| --- | --- | --- | --- | --- | --- | --- | --- | --- | --- |
|  |  | VIP | P-Value | Fold Change | Trend | VIP | P-Value | Fold Change | Trend |
| 1 | Pyrimidine | 1.90 | 0.014 | 0.635 | Down | 2.30 | 0.01 | 1.61 | Up |
| 2 | Thiamine | 1.20 | 0.04 | 0.61 | Down | 1.38 | 0.02 | 1.70 | Up |
| 3 | 5-(2-Hydroxyethyl)-4-methylthiazole | 1.22 | 0.038 | 0.63 | Down | 1.52 | 0.01 | 1.76 | Up |
| 4 | 3-Formyl-6-hydroxyindole | 1.74 | 0.01 | 0.60 | Down | 1.59 | 0.048 | 1.54 | Up |
| 5 | Acetone cyanohydrin | 1.54 | 0.027 | 0.66 | Down | 1.79 | 0.03 | 1.55 | Up |
| 6 | 1-Methyladenine | 1.90 | 0.01 | 0.68 | Down | 2.00 | 0.027 | 1.46 | Up |
| 7 | Benzaldehyde | 1.33 | 0.005 | 0.52 | Down | 1.58 | 0.017 | 2.27 | Up |
| 8 | 5-Aminopentanal | 1.57 | 0.024 | 0.74 | Down | 1.74 | 0.02 | 1.39 | Up |
| 9 | Dimethylglycine | 1.82 | 0.039 | 0.77 | Down | 1.69 | 0.05 | 1.26 | Up |
| 10 | 2-(Methylamino)benzoic acid | 2.22 | 0.001 | 0.67 | Down | 2.11 | 0.012 | 1.37 | Up |
| 11 | Aminoadipic acid | 1.45 | 0.01 | 0.604 | Down | 1.43 | 0.041 | 1.56 | Up |
| 12 | Toluene | 1.88 | 0.042 | 0.843 | Down | 1.94 | 0.037 | 1.25 | Up |
